# Cost-effectiveness of Screening for Atrial Fibrillation Using Wearable Devices

**DOI:** 10.1001/jamahealthforum.2022.2419

**Published:** 2022-08-05

**Authors:** Wanyi Chen, Shaan Khurshid, Daniel E. Singer, Steven J. Atlas, Jeffrey M. Ashburner, Patrick T. Ellinor, David D. McManus, Steven A. Lubitz, Jagpreet Chhatwal

**Affiliations:** 1 Institute for Technology Assessment, Massachusetts General Hospital, Boston; 2 Department of Radiology, Harvard Medical School, Boston, Massachusetts; 3 Cardiovascular Research Center, Massachusetts General Hospital, Boston; 4 Demoulas Center for Cardiac Arrhythmias, Massachusetts General Hospital, Boston; 5 Division of General Internal Medicine, Massachusetts General Hospital, Boston; 6 Department of Medicine, Harvard Medical School, Boston, Massachusetts; 7 Department of Medicine, University of Massachusetts Chan Medical School, Worcester

## Abstract

**Importance:**

Undiagnosed atrial fibrillation (AF) is an important cause of stroke. Screening for AF using wrist-worn wearable devices may prevent strokes, but their cost-effectiveness is unknown.

**Objective:**

To evaluate the cost-effectiveness of contemporary AF screening strategies, particularly wrist-worn wearable devices.

**Design, Setting, and Participants:**

This economic evaluation used a microsimulation decision-analytic model and was conducted from September 8, 2020, to May 23, 2022, comprising 30 million simulated individuals with an age, sex, and comorbidity profile matching the US population aged 65 years or older.

**Interventions:**

Eight AF screening strategies, with 6 using wrist-worn wearable devices (watch or band photoplethysmography, with or without watch or band electrocardiography) and 2 using traditional modalities (ie, pulse palpation and 12-lead electrocardiogram) vs no screening.

**Main Outcomes and Measures:**

The primary outcome was the incremental cost-effectiveness ratio, defined as US dollars per quality-adjusted life-year (QALY). Secondary measures included rates of stroke and major bleeding.

**Results:**

In the base case analysis of this model, the mean (SD) age was 72.5 (7.5) years, and 50% of the individuals were women. All 6 screening strategies using wrist-worn wearable devices were estimated to be more effective than no screening (range of QALYs gained vs no screening, 226-957 per 100 000 individuals) and were associated with greater relative benefit than screening using traditional modalities (range of QALYs gained vs no screening, −116 to 93 per 100 000 individuals). Compared with no screening, screening using wrist-worn wearable devices was associated with a reduction in stroke incidence by 20 to 23 per 100 000 person-years but an increase in major bleeding by 20 to 44 per 100 000 person-years. The overall preferred strategy was wearable photoplethysmography, followed conditionally by wearable electrocardiography with patch monitor confirmation, which had an incremental cost-effectiveness ratio of $57 894 per QALY, meeting the acceptability threshold of $100 000 per QALY. The cost-effectiveness of screening was consistent across multiple scenarios, including strata of sex, screening at earlier ages (eg, ≥50 years), and with variation in the association of anticoagulation with risk of stroke in the setting of screening-detected AF.

**Conclusions and Relevance:**

This economic evaluation of AF screening using a microsimulation decision-analytic model suggests that screening using wearable devices is cost-effective compared with either no screening or AF screening using traditional methods.

## Key Points

**Question:**

Is population-based atrial fibrillation (AF) screening using wearable devices cost-effective?

**Findings:**

In this economic evaluation of 30 million simulated individuals with an age, sex, and comorbidity profile matching the US population aged 65 years or older, AF screening using wearable devices was cost-effective, with the overall preferred strategy identified as wearable photoplethysmography, followed conditionally by wearable electrocardiography with patch monitor confirmation (incremental cost-effectiveness ratio, $57 894 per quality-adjusted life-year). The cost-effectiveness of screening was consistent across multiple scenarios, including strata of sex, screening at earlier ages, and with variation in the association of anticoagulation with risk of stroke associated with screening-detected AF.

**Meaning:**

This study suggests that contemporary AF screening using wearable devices may be cost-effective.

## Teaser

This economic evaluation uses a microsimulation decision-analytic model to assess the cost-effectiveness of contemporary atrial fibrillation screening strategies, particularly wrist-worn wearable devices.

## Introduction

Undiagnosed atrial fibrillation (AF) is an important cause of stroke.^1^ Oral anticoagulation (OAC) can prevent AF-related strokes.^2^ Population-based AF screening may facilitate earlier initiation of OAC treatment to prevent stroke, but improved outcomes have not been clearly demonstrated.^3,4,5,6^ Most conventional AF screening strategies studied to date have used single time-point screening,^7,8,9^ which may be limited in the detection of paroxysmal AF. More recently, it has become possible to perform screening using wrist-worn wearable devices, which may facilitate repeated screening with greater sampling density and over longer durations to detect less-frequent AF episodes. However, longer screening durations may lead to increased costs and harms associated with downstream testing and false positives.^10,11^ False positives may be amplified by disproportionate screening of younger individuals, who are more likely to use wearable devices and less likely to have AF.^12^ The association between very infrequent AF episodes and stroke risk is less clear.^10^

Given the uncertainty regarding the effectiveness of AF screening, current guidelines offer conflicting endorsements. Cardiology societies from Europe^13^ and Australia or New Zealand^14^ provide a class I recommendation for opportunistic screening using pulse palpation or electrocardiography (ECG) rhythm strip for individuals 65 years of age or older. In contrast, the US Preventive Services Task Force has concluded that there is insufficient evidence for or against AF screening using ECG.^15^ Logistical constraints may render randomized clinical trials comparing the myriad potential AF screening strategies impractical. For example, many consumers are increasingly adopting wearable devices,^16^ potentially contaminating any randomization approach, and trials assessing hard end points, such as stroke, require exceptionally large samples for adequate power.

We therefore developed a simulation model to flexibly compare multiple AF screening strategies, including both traditional modalities (eg, 12-lead ECG [12L ECG]) and wrist-worn wearable devices. A previous study using a related model demonstrated that screening via wearable devices may decrease AF-related stroke on a population level.^11^ Because mass AF screening may lead to increased health care use and associated costs, however, it is imperative to assess whether the benefits of screening come at a cost acceptable to potential payers.

In the present study, the cost-effectiveness of 8 AF screening strategies, including 6 using wrist-worn wearable devices, was compared with no screening. The model adopted a health care system perspective and a screening regimen under the hypothetical scenario that a clinician would prescribe the wearable device.

## Methods

### Model Overview

The general structure of our AF screening simulation model has been described previously.^11^ In brief, the clinical course of AF was modeled using a patient-level state-transition approach, implemented using C++. The model simulated a 30 million–person cohort matched on age and comorbidity distribution with the 2019 US population aged 65 years or older—the age at which AF screening is recommended by guidelines.^13,14^ A 50%-50% distribution of sexes was assumed, approximating the US sex distribution. Only individuals at sufficient risk of stroke to merit OAC treatment based on the CHA_2_DS_2_-VASC (cardiac failure or dysfunction, hypertension, age 65-74 [1 point] or ≥75 years [2 points], diabetes, and stroke, transient ischemic attack, or thromboembolism [2 points]–vascular disease, and sex category [female]) risk score^17,18^ in the presence of an AF diagnosis were screened. The time between state transitions was 1 month, and the simulation continued until death or 100 years of age. Given our intent to compare very different AF screening strategies, including those using wearable devices (which have not been assessed in randomized clinical trials, to our knowledge), the association of screening with outcomes was estimated using population-based estimates of event rates and treatment effects, as well as the test characteristics of the screening modalities used, while accounting for uncertainty in model estimates. Model development and reporting were performed in accordance with the Consolidated Health Economic Evaluation Reporting Standards (CHEERS) reporting guideline. The analysis was conducted from September 8, 2020, to May 23, 2022. The study was exempt from institutional review board review because it used only publicly available data and was not human participants research. Informed consent was not necessary because simulated individuals were used.

### Screening Strategies

**Figure 1.**
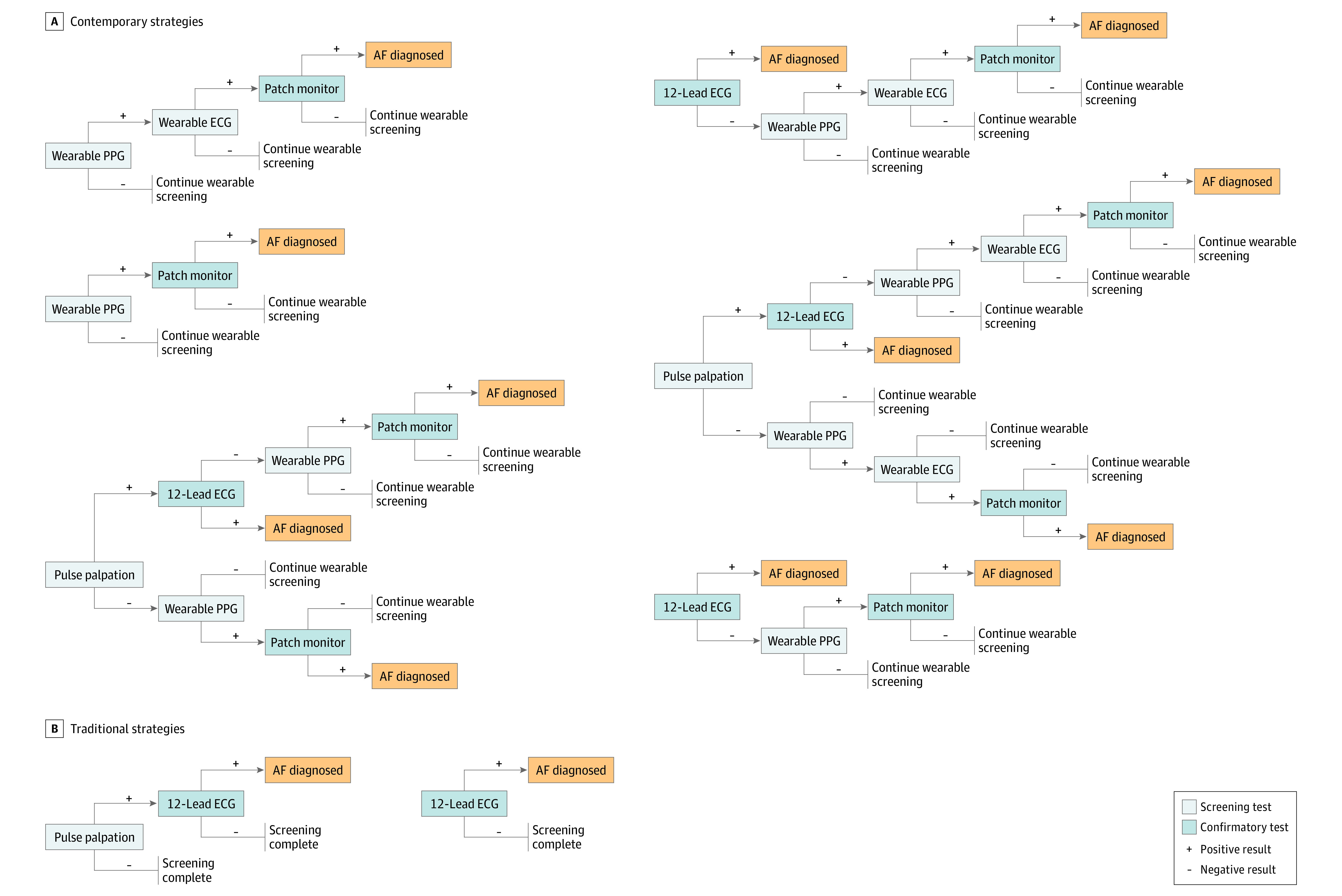
Simulated Atrial Fibrillation Screening Strategies Eight atrial fibrillation (AF) screening strategies evaluated in our cost-effectiveness model are shown. Six contemporary strategies use a wearable device in the screening pathway. Two established traditional screening strategies were included for comparison. ECG indicates electrocardiography; PPG, photoplethysmography.

The simulation included no screening in addition to 8 AF screening strategies. Six strategies using wrist-worn wearable devices were modeled, with strategies chosen based on evidence of clinical effectiveness in a prior analysis,^11^ as well as 2 traditional strategies previously studied in randomized clinical trials of AF screening^8^ as a comparison (ie, pulse palpation with confirmatory 12L ECG and 12L ECG alone). An overview of all strategies is shown in Figure 1.

As performed previously,^11^ we assessed wearable devices with photoplethysmography (PPG) with or without reflexive single-lead ECG (1L ECG) capability. For wearable devices with both PPG and 1L ECG capability, PPG was the default function and operated passively throughout the wear time. Single-lead ECG would be triggered only after abnormal PPG signals. Discrete modalities were those capable only of instantaneous AF detection (eg, pulse palpation, 12L ECG), and confirmatory tests were those performed conditionally after an abnormal result on a previous test. Lifetime screening was modeled for wearable devices, with adjustment for clinical attrition rates and incomplete wear time. All positive wearable signals were assumed to require confirmation with 2-week ECG patch monitor (Figure 1) prior to an AF diagnosis being made. Individuals were assumed to adhere to the screening strategy recommended by a clinician, but with consideration of wearable device attrition. There was no crossover among strategies and no background wearable use in the no-screening strategy. Key model parameters are shown in Table 1.^19,20,21,22,23,24,25,26,27,28,29,30,31,32,33,34,35,36,37,38,39,40,41,42,43,44,45^

**Table 1.** Key Clinical Parameters

Parameter	Base case (lower-upper estimates)	References
AF		
Proportion of AF that is undiagnosed	0.24 (0.22-0.28)	^19,20^
Proportion of undiagnosed AF that is persistent	0.41 (0.04-0.66)	^20,21,22,23^
Mean AF burden in individuals with paroxysmal AF, %	4.5 (1.1-17.0)	^20,24,25^
Relative risk of ischemic stroke for paroxysmal screening-detected AF (vs persistent AF)^a^	1.00 (0.75-1.00)	^26^ (Assumption)
Patient factors		
Proportion of OAC that is NOAC (vs warfarin)	0.33 (0.10-0.50)	^27,28^
Yearly probability of warfarin discontinuation	0.10 (0.08-0.40)	^29,30^
Relative risk of NOAC discontinuation (vs warfarin)	0.69 (0.57-0.84)	^29^
Initial uptake of follow-up patch monitoring, %	100 (62-100)	^31,32^ (Assumption)
Ischemic stroke		
Proportion of strokes that are ischemic	0.87 (0.83-0.88)	^33^
Relative risk of ischemic stroke		
Aspirin vs placebo, AF	0.78 (0.65-0.94)	^34^
Warfarin vs placebo, AF	0.33 (0.23-0.46)	^34^
OAC vs placebo, no AF	0.58 (0.44-0.76)	^35^
NOAC vs warfarin	1 (0.83-1.02)	^36^
OAC plus aspirin vs OAC alone	1 (0.44-2.22)	^37^
Screening methods		
Sensitivity (single time point), %		
Pulse palpation	89.0 (16-100)	^8,38,39^
Single-lead handheld ECG	96.9 (36.8-100)	^40,41^
Patch monitor	100 (90-100)	^42^
12-Lead ECG	90.0 (52-100)	^43^
Smartwatch or band		
PPG^b^	95.3 (92-97.4)	^31,44^
ECG	85.2 (76.7-98.3)	^45^
Specificity (single time point), %		
Pulse palpation	81.0 (65-91)	^8,38,39^
Single-lead handheld ECG	89.6 (71-100)	^40^
Patch monitor	96.6 (86.9-100)	^42^
12-Lead ECG	98.3 (55-100)	^43^
Smartwatch or band		
PPG^b^	99.7 (98.1-99.9)	^31,44^
ECG^b^	99.6 (89.6-100)	^45^

Abbreviations: AF, atrial fibrillation; ECG, electrocardiography; NOAC, novel oral anticoagulant; OAC, oral anticoagulation; PPG, photoplethysmography.

^a^ Range, 1% to 100% additionally assessed in dedicated 1-way sensitivity analyses.

^b^ Values of 85% and 80% additionally assessed in dedicated scenario analyses.

### Events and Utilities

Rates of AF, stroke, and bleeding were obtained from the published literature.^11^ Associations of antithrombotic therapy with AF-related stroke were similarly estimated. In the primary model, paroxysmal and persistent AF conferred similar stroke risk, but because the association between AF burden and stroke risk remains incompletely characterized, we performed sensitivity analyses, varying the relative risk of stroke associated with paroxysmal AF between 1% and 100% of that observed with persistent AF.^24,46,47^ Adherence to OAC treatment was 100% at AF diagnosis, but discontinuation was modeled using clinical OAC discontinuation rates.^29,30^ Sensitivity analyses were performed to assess the association with outcomes of incomplete OAC uptake (60%-100%). Utilities were estimated for all major events, including stroke, intracranial hemorrhage, and major bleeding, stratified by severity. Treatment-associated utilities were also modeled. All utilities were derived from the published literature.

### Costs

Costs were modeled from the health care system perspective. One-time event costs were applied for ischemic stroke, intracranial hemorrhage, and bleeding events, stratified by severity. Estimates were obtained from the Agency for Healthcare Research and Quality Healthcare Cost and Utilization Project reflecting US hospitalization data from 2016.^48^ Long-term event costs were obtained from the published literature and stratified by severity. Treatment-related costs included primary drug costs and costs associated with regular international normalized ratio testing for individuals taking warfarin and annual physician visits for all individuals receiving OAC. Screening-related costs included direct costs associated with the modalities used (eg, cost of patch monitor), nurse visit costs at screening administration, and physician visit costs after each new AF diagnosis (ie, true positive or false positive). For strategies using wrist-worn wearable devices, the manufacturer-suggested retail price of the device was applied as the direct cost. Devices were assumed to require replacement after every 5 years of consecutive screening. Physician and nurse professional fees were obtained from the Centers for Medicare & Medicaid Services.^49^ All other costs were extracted from the published literature. Cost estimates were adjusted for inflation and converted to US dollars as of June 2020. All event rate, utility, and cost parameters are listed in eTables 1 to 7 in the Supplement.

### Cost-effectiveness

For each simulated strategy, total quality-adjusted life-years (QALYs), total costs, and incremental cost-effectiveness ratios (ICERs) were calculated. All future QALYs and costs were discounted at 3% per year. To identify the optimal cost-effective strategy, a willingness-to-pay threshold of $100 000 per QALY was applied.^50,51^

### Scenario Analyses

Multiple scenario analyses were performed to assess the cost-effectiveness of screening across subgroups of clinical interest. Specifically, 3 scenarios for the age distribution of the cohort (≥50, ≥55, and ≥60 years) were assessed, as a surrogate for the age at which AF screening begins. In addition, screening was modeled among only women and separately among only men. Three scenarios for the mean daily wear time of the wrist-worn wearable devices (6, 12, and 24 hours) and 4 scenarios for the cost of the wrist-worn wearable devices ($150, $200, $250, and $300) were also assessed.

### Uncertainty Analyses

The association of parameter uncertainty of the strategies with the cost-effectiveness frontier and their corresponding ICERs was assessed using sensitivity analysis. In 1-way sensitivity analyses, a subset of all model parameters chosen based on economic importance, clinical salience, or demonstration of influence in previously published models (eTable 8 in the Supplement) was varied across evidence-based ranges.^52,53^ In probabilistic sensitivity analyses, all model parameters were varied according to their corresponding random distributions across the same ranges (eTable 8 in the Supplement). Because uncertainty was modeled using probabilistic analyses, formal statistical testing was not used.

## Results

### Base Case Analyses

In the base case analysis, the mean (SD) age was 72.5 (7.5) years, and 50% of the individuals were women. No screening resulted in 7.092 QALYs per individual. In total, 7 of 8 strategies (87.5%) resulted in additional QALYs compared with no screening (93-957 QALYs gained per 100 000 persons). The single strategy that provided lower QALYs vs no screening was 1-time 12L ECG screening (7.090 QALYs per person). The strategy providing the greatest QALYs was a sequence of wrist-worn wearable PPG followed conditionally by wrist-worn wearable 1L ECG and confirmatory patch monitor (7.101 QALYs per person). All 6 screening strategies involving wrist-worn wearable devices were estimated to be more effective than no screening (226-957 QALYs gained per 100 000 persons) and were associated with greater relative benefit than screening using traditional modalities vs no screening (−116 to 93 QALYs gained per 100 000 persons). Strategies using wrist-worn wearable devices were associated with reduction in stroke incidence by 20 to 23 stroke events per 100 000 person-years but an increase in major bleeding by 20 to 44 major bleeding events per 100 000 person-years.

Compared with no screening, screening using pulse palpation and 12L ECG was cost saving ($43 per person). All other strategies incurred additional costs ($61-$603 per person), with strategies using wrist-worn wearable devices being more costly ($458-$603 per person) than 12L ECG alone ($61 per person screened). Excess costs were primarily associated with direct screening costs (eg, cost of the wrist-worn wearable device) and use of OAC (eTable 9 in the Supplement).

**Figure 2.**
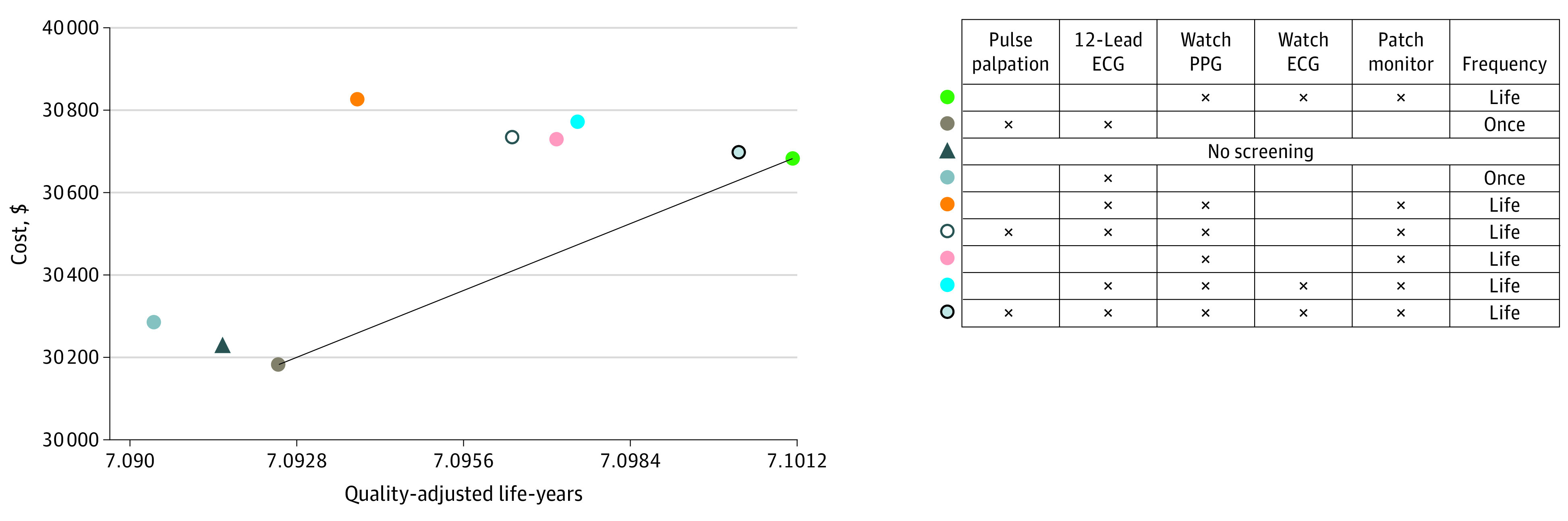
Cost-effectiveness Frontier of Simulated Atrial Fibrillation Screening Strategies Each point indicates the combination of effectiveness and cost of a given strategy. The slope connecting 2 points indicates the incremental cost-effectiveness ratio (ICER), a summary measure of cost-effectiveness, representing the ratio of the incremental cost vs the incremental effectiveness associated with going from 1 strategy to another. The diagonal line indicates the ICER of the most cost-effective strategy (wrist-worn wearable photoplethysmography [PPG] followed conditionally by wrist-worn wearable single-lead electrocardiography [ECG] and confirmatory patch monitor; ICER, $57 894). Every row in the table to the right represents a given strategy. For each row, an X indicates that a given modality was included in the screening strategy. Absence of an X indicates that a given modality was not included.

In terms of cost-effectiveness, pulse palpation and 12L ECG dominated no screening ($43 saved per person; 93 QALYs gained per 100 000 persons). The overall cost-effective strategy was wrist-worn wearable PPG followed conditionally by wrist-worn wearable 1L ECG and confirmatory patch monitor (ICER, $57 894 per QALY) (Table 1^19,20,21,22,23,25,26,27,28,31,32,33,34,35,36,37,38,39,40,41,42,43,44,45^ and Figure 2), which had a favorable false-positive rate of 0.4% (eTable 10 in the Supplement).

### Scenario Analyses

The relative cost-effectiveness of the AF screening strategies modeled was generally consistent across the 12 clinical scenarios considered. Specifically, 1-time screening using pulse palpation and 12L ECG consistently dominated no screening, and the most cost-effective strategy was consistently wrist-worn wearable PPG followed conditionally by wrist-worn wearable 1L ECG and confirmatory patch monitor (ICER range, $34 583-$90 909 per QALY).

Across scenarios of target age distribution (Table 2), the ICER of the most cost-effective strategy increased as the minimum age of the cohort decreased starting with individuals aged 60 years or older (individuals aged ≥60 years: $59 143 per QALY; individuals aged ≥50 years: $90 909 per QALY), although screening remained cost-effective even at the lowest target age evaluated (≥50 years). Similarly, the ICER of the most cost-effective strategy increased as the cost of the wrist-worn wearable increased from $150 to $300 ($150: $34 583 per QALY; $300: $76 956 per QALY) and the mean daily wear time decreased from 24 hours to 6 hours (24 hours: $59 288 per QALY; 6 hours: $56 314 per QALY), but increases were generally modest (eTable 11 in the Supplement). Screening with the preferred strategy also remained cost-effective when targeting only women (ICER, $57 340 per QALY) and separately only men (ICER, $60 375 per QALY). Cost-effectiveness also persisted when values as low as 80% were assumed for wearable sensitivity and specificity (ICER: 80% with PPG, $57 874 per QALY; 80% with ECG, $69 891 per QALY). Details of scenario analyses are shown in eTable 11 in the Supplement.

**Table 2.** Cost-effectiveness Results in the Base Case and Across Varying Age Thresholds^a^

Strategy						QALYs	Cost, $	Incremental cost-effectiveness ratio, $/QALY
PP	12L ECG	PPG	1L ECG	PM	Frequency			
**Aged ≥65 y (base case)**								
X	X				Once	7.09	30 182	[Reference]
		X	X	X	Life	7.10	30 683	57 894
No screening						7.09	30 225	Strongly dominated
	X				Once	7.09	30 286	Strongly dominated
	X	X		X	Life	7.09	30 828	Strongly dominated
X	X	X		X	Life	7.10	30 735	Strongly dominated
		X		X	Life	7.10	30 730	Strongly dominated
	X	X	X	X	Life	7.10	30 772	Strongly dominated
X	X	X	X	X	Life	7.10	30 698	Strongly dominated
**Aged ≥60 y**								
X	X				Once	8.26	31 396	[Reference]
		X	X	X	Life	8.27	31 927	59 143
No screening						8.26	31 410	Strongly dominated
	X				Once	8.25	31 500	Strongly dominated
	X	X		X	Life	8.26	32 118	Strongly dominated
X	X	X		X	Life	8.26	31 991	Strongly dominated
		X		X	Life	8.26	32 009	Strongly dominated
	X	X	X	X	Life	8.26	32 035	Strongly dominated
X	X	X	X	X	Life	8.27	31 914	Weakly dominated
**Aged ≥55 y**								
X	X				Once	9.35	29 813	[Reference]
		X	X	X	Life	9.36	30 448	76 889
No screening						9.35	29 855	Strongly dominated
	X				X	9.35	29 960	Strongly dominated
	X	X			X	9.35	30 651	Strongly dominated
X	X	X		X	X	9.35	30 537	Strongly dominated
		X				9.36	30 527	Strongly dominated
	X	X	X		X	9.35	30 603	Strongly dominated
X	X	X	X	X	X	9.36	30 458	Strongly dominated
**Aged ≥50 y**								
X	X				Once	10.31	28 538	[Reference]
No screening						10.31	28 555	57 333
		X	X	X	Life	10.32	29 255	90 909
	X				Once	10.30	28 683	Strongly dominated
	X	X		X	Life	10.30	29 433	Strongly dominated
X	X	X		X	Life	10.31	29 285	Strongly dominated
		X		X	Life	10.31	29 318	Strongly dominated
	X	X	X	X	Life	10.31	29 389	Strongly dominated
X	X	X	X	X	Life	10.32	29 261	Strongly dominated

Abbreviations: PM, patch monitor; PP, pulse palpation; PPG, photoplethysmography; QALYs, quality-adjusted life-years; 1L ECG, single-lead electrocardiogram; 12L ECG, 12-lead electrocardiogram.

^a^ Every row represents a given strategy. For each row, an X indicates that a given modality was included in the screening strategy. Absence of an X indicates that a given modality was not included.

### Sensitivity Analyses

One-way sensitivity analyses were broadly consistent with base case results. Specifically, wrist-worn wearable PPG followed conditionally by wrist-worn wearable 1L ECG and confirmatory patch monitor remained the most cost-effective strategy in 94% of scenarios (ICER range, $47 822-$71 576 per QALY) (eTable 12 in the Supplement). Modality specificity was particularly associated with cost-effectiveness. For example, at lower 12L ECG specificity (76%), 1-time screening using pulse palpation and 12L ECG was no longer cost-effective. At a higher wrist-worn PPG specificity (100%), wrist-worn wearable PPG followed conditionally by patch monitor alone (ie, without reflexive 1L ECG) was preferred (ICER, $56 610 per QALY). The relative risk of stroke given paroxysmal vs persistent AF was also associated with cost-effectiveness, whereby wearable screening remained the preferred strategy until stroke risk with paroxysmal AF decreased less than 5% of that observed with persistent AF, at which point traditional screening with pulse palpation followed by 12L ECG became the preferred strategy. Wearable screening remained cost-effective even as initial OAC uptake varied between 60% and 100%. All 1-way sensitivity analyses are presented in eTable 12 in the Supplement.

**Figure 3.**
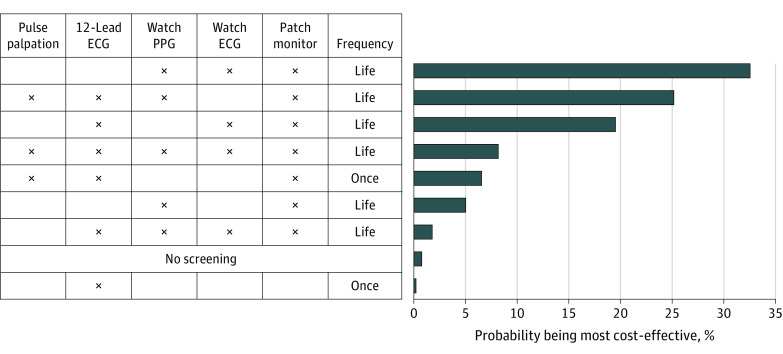
Probabilistic Sensitivity Analysis Each bar indicates the probability that a given strategy is cost-effective, when accounting for parameter uncertainty. Strategies are displayed in order of decreasing probability of cost-effectiveness, with the strategy most likely to be cost-effective at the top. Every row in the table to the left represents a given strategy. For each row, an X indicates that a given modality was included in the screening strategy. Absence of an X indicates that a given modality was not included. ECG indicates electrocardiography; PPG, photoplethysmography.

Probabilistic sensitivity analyses demonstrated moderate changes in cost-effectiveness associated with parameter uncertainty. The preferred strategy in the base case remained cost-effective in the plurality (33%) of simulations (Figure 3). Two strategies emerged as cost-effective in a considerable proportion of simulations, specifically pulse palpation and 12L ECG followed conditionally by wrist-worn wearable PPG with confirmatory patch monitor (25% of simulations) and 12L ECG followed conditionally by wrist-worn wearable PPG and confirmatory patch monitor (20% of simulations). Cost-effectiveness acceptability curves are shown in eFigure in the Supplement.

## Discussion

With the use of a comprehensive economic model to simulate 30 million individuals matching the US population with a guideline-based indication for AF screening (ie, aged ≥65 years),^13,14^ AF screening using wearable devices was estimated to be cost-effective. Specifically, of 8 strategies evaluated in addition to no screening, the economically preferred strategy was wrist-worn wearable PPG followed conditionally by wrist-worn wearable 1L ECG with patch monitor confirmation, having an ICER just below $60 000 per QALY. In scenario analyses, wearable screening remained cost-effective across multiple conditions, including targeting individuals at younger ages than currently endorsed by guidelines (eg, ≥50 years).^13,14^ On balance, our findings suggest that AF screening using wearable devices may be cost-effective.

Our study extends previous findings by comparing the cost-effectiveness of AF screening using both traditional modalities and wearable devices. Aronsson et al^47^ previously estimated that AF screening with handheld 1L ECG twice daily over 2 weeks is cost-effective among individuals aged 75 years or older (ICER, €7855 per QALY) as well as among individuals aged 65 years or older (ICER, €24 500 per QALY).^54^ Given our focus on wearable devices, we did not directly evaluate handheld 1L ECG, although we observed that a similar combination of pulse palpation followed by 12L ECG was on the cost-effectiveness frontier with comparable ICERs. More recently, Oguz et al^55^ found that 1-time screening using 12L ECG alone followed by 14-day screening with patch monitor at 75 years of age was also cost-effective. In general, our findings support previous observations suggesting that AF screening can be cost-effective, and they provide new evidence that strategies using wearable devices may be economically favorable.

Our results provide justification for the potential use of wrist-worn wearable devices integrated into composite AF screening pathways. The use of wearable devices for AF screening is expanding,^16^ but their optimal role within an AF screening program is unclear. The most cost-effective strategy that we identified was wrist-worn wearable PPG followed conditionally by wrist-worn wearable 1L ECG and confirmatory patch monitor, providing a net benefit at $57 894 per QALY. The use of wrist-worn wearable devices over years may facilitate an extended duration of screening, which may lead to detection of infrequent paroxysmal AF that may be otherwise difficult to ascertain. At the same time, the use of patch monitor confirmation of abnormal signals from wrist-worn wearable devices reduced false positives. Wearable screening remained cost-effective even as analyzable wear time decreased to 6 hours per day,^56^ suggesting that wearable devices may remain effective even if wear time is incomplete or limited to certain periods (eg, sleep). Assessment of contemporary screening pathways, including wearable devices applied over many years, may account for differences between our results and the results of recent trials demonstrating little or no effect of discrete screening on short-term AF diagnosis.^4,7,9^ The preferred screening pathway began directly with wearable devices, suggesting that screening initiated at the level of the consumer device may be economically favorable. Randomized clinical trials are warranted to better quantify potential benefits of AF screening using wearable devices, and our findings may be useful to inform the design of such trials to optimize clinical and economic efficiency.

The cost-effectiveness of AF screening pathways may be maximized by using scalable, highly sensitive screening methods followed by confirmatory testing. Both screening strategies appearing on the cost-effectiveness frontier included a highly sensitive modality upfront (eg, pulse palpation, wearable PPG), followed by a confirmatory test (eg, 12L ECG, patch monitor). Use of a sensitive modality likely functions to rule out most individuals without AF with relatively modest resource use. At the same time, confirmatory testing prevents excess costs and harm associated with false positives. Our results suggest that the optimal combination of modalities may depend on the resources available to the health care system, with wrist-worn wearable screening and confirmatory patch monitor identified as the preferred strategy at the $100 000 per QALY threshold, whereas pulse palpation and 12L ECG may be preferable in lower-resource settings. Because health care costs are relatively high in the US and vary across settings,^57^ future analyses of other health care systems may be useful to quantify the cost-effectiveness of AF screening using wearable devices in alternative settings.

Our analyses suggest that contemporary AF screening remains cost-effective across a range of scenarios and may even extend to populations not currently considered for AF screening. Wearable screening remained cost-effective across strata of sex and substantial variations in wearable device–related costs and mean daily wear time. Cost-effectiveness of wearable screening also persisted when assuming values as low as 80% for the sensitivity and specificity of wearable modalities, likely owing to the use of patch monitor confirmation. In analyses varying the target age for screening, we observed continued cost-effectiveness even as screening was extended to individuals aged 50 years or older with risk factors for stroke. On balance, such findings suggest that AF screening may be effective and economically acceptable even among individuals below the guideline-based age threshold of 65 years who have risk factors for AF-related stroke.^13,14^

### Limitations

This study has some limitations. First, this is a simulation study using population-based estimates to infer the cost-effectiveness of several AF screening strategies, some of which have not been assessed in randomized clinical trials. Nevertheless, our results are consistent with previous analyses of traditional screening modalities^14,47^ and remained robust across wide variation in model estimates to account for uncertainty. Second, the test characteristics of contemporary AF screening modalities are less well known than those for more established modalities. As a result, we varied all test characteristics over plausible ranges in sensitivity analyses. Third, to quantify the relative associations of each AF screening strategy with outcomes, we assumed no background wearable technology use in the no-screening strategy. Current data on the use of wearable devices, particularly among the older age groups being simulated, are too limited to accurately model.^12^ Fourth, we assumed in our base model that paroxysmal AF and persistent AF were associated with similar stroke risk, although recent evidence suggests that AF burden may be significantly associated with stroke.^10^ Nevertheless, we found that wearable screening remained cost-effective even when the relative risk of stroke with paroxysmal AF was only 5% that of persistent AF (ie, assuming a larger association between AF burden and stroke than previously reported).^10,58^ Fifth, a large simulation size and longer screening duration allowed us to detect potential associations of long-term AF screening with stroke, which, although clinically significant on a population scale, may be challenging to demonstrate in a traditional clinical trial.^6,7,59^ Therefore, although our results cannot be considered definitive, they prioritize specific wearable screening strategies as likely to be cost-effective, thereby meriting dedicated prospective study.

## Conclusions

This modeling study of 8 contemporary AF screening strategies suggests that specific forms of AF screening using wearable devices may be cost-effective. The cost-effectiveness of AF screening enabled by wearable devices persisted across multiple clinically relevant scenarios, including screening a general population aged 50 years or older with risk factors for stroke. When deployed within specific AF screening pathways, wearable devices are likely to be an important component of cost-effective AF screening.

## Notes

Supplement. **eMethods.** Supplemental Methods **eTable 1.** Disease Incidence (per 1000 Person-Years) **eTable 2.** Comorbidity Prevalence/Incidence (per 1000 Person-Years) **eTable 3.** Disease Recurrence Rates (Monthly Probabilities) **eTable 4.** Disease-Related Mortality (Monthly Probabilities) **eTable 5.** Severity Measures **eTable 6.** Utilities **eTable 7.** Costs **eTable 8.** Summary of Parameters Included in Sensitivity Analyses **eTable 9.** Summary of Costs Associated With Each Screening Strategy **eTable 10.** True and False Positive Rates by Strategy **eTable 11.** Cost-effectiveness Results for Scenario Analyses **eTable 12.** One-Way Sensitivity Analysis **eFigure.** Cost-effectiveness Acceptability Curves
eReferences.
