# Supplementary material for: Cost-effectiveness of Screening for Atrial Fibrillation Using Wearable Devices
Source: JAMA Health Forum. 2022 Aug 5;3(8):e222419. doi: 10.1001/jamahealthforum.2022.2419 (PMC9356321; doi:10.1001/jamahealthforum.2022.2419)
Supplement: Supplement. — eMethods. Supplemental Methods eTable 1. Disease Incidence (per 1000 Person-Years) eTable 2. Comorbidity Prevalence/Incidence (per 1000 Person-Years) eTable 3. Disease Recurrence Rates (Monthly Probabilities) eTable 4. Disease-Related Mortality (Monthly Probabilities) eTable 5. Severity Measures eTable 6. Utilities eTable 7. Costs eTable 8. Summary of Parameters Included in Sensitivity Analyses eTable 9. Summary of Costs Associated With Each Screening Strategy eTable 10. True and False Positive Rates by Strategy eTable 11. Cost-effectiveness Results for Scenario Analyses eTable 12. One-Way Sensitivity Analysis eFigure. Cost-effectiveness Acceptability Curves eReferences. [file jamahealthforum-e222419-s001.pdf]

Supplementary Online Content

Chen W, Khurshid S, Singer DE, et al. Cost-effectiveness of screening for atrial fibrillation using wearable devices. *JAMA Health Forum.* 2022;3(8):e222419. doi:10.1001/jamahealthforum.2022.2419

eMethods. Supplemental Methods
eTable 1. Disease Incidence (per 1000 Person-Years)
eTable 2. Comorbidity Prevalence/Incidence (per 1000 Person-Years)
eTable 3. Disease Recurrence Rates (Monthly Probabilities)
eTable 4. Disease-Related Mortality (Monthly Probabilities)
eTable 5. Severity Measures
eTable 6. Utilities
eTable 7. Costs
eTable 8. Summary of Parameters Included in Sensitivity Analyses
eTable 9. Summary of Costs Associated With Each Screening Strategy
eTable 10. True and False Positive Rates by Strategy
eTable 11. Cost-effectiveness Results for Scenario Analyses
eTable 12. One-Way Sensitivity Analysis
eFigure. Cost-effectiveness Acceptability Curves
eReferences.

This supplementary material has been provided by the authors to give readers additional information about their work.

© 2022 Chen W et al. *JAMA Health Forum.*

eMethods. Supplemental Methods

Event-related costs
For clinical events modeled (e.g., ischemic stroke, intracranial hemorrhage, and major bleeding), upfront costs were stratified by severity and obtained from the Agency for Healthcare Research and Quality (https://hcupnet.ahrq.gov/#setup) as follows: First, we extracted separate cost statistics for all International Classification of Diseases, 10th revision (ICD-10) diagnosis codes corresponding to the event of interest. Then, we sorted the costs in ascending order and divided them into quantiles equal in number to the categories of severity (e.g., tertiles for mild/moderate/severe groupings). Within each quantile, we utilized the mean hospital cost as the base case cost for the event at the corresponding severity level. The lower and upper bounds were set as the minimum and maximum cost values observed within the quantile.

In cases where one has multiple competing event-related costs, either the most relevant cost is incurred, or the maximum of the costs is incurred. For example, a history of stroke is associated with a maintenance cost associated with chronic post-stroke care. If a recurrent acute stroke occurs, only the upfront cost corresponding to the new stroke is invoked (since it is greater than the maintenance cost associated with chronic post-stroke care), with no additional maintenance cost.

Drug/visit costs
In cases where anticoagulation was stopped due to a history of major bleeding, or in accordance with modeled discontinuation rates, we assumed that the monthly drug cost would stop accumulating until the treatment regimen was resumed. We also assumed that physician visits for acute events (e.g., major bleeding) would also fulfill potential maintenance visit requirements. For example, if an individual on anticoagulation has a physician visit secondary to an acute bleed, that individual's next annual physician visit for anticoagulation maintenance would be no less than one year after the acute bleed.

Screening costs
For discrete screening modalities, namely single-lead ECG, 12-lead ECG, pulse palpation, and patch monitor, a one-time screening cost was incurred if and only if the test was performed.

For costs associated wrist-worn wearable screening, a one-time upfront cost was incurred upon the start of screening (corresponding to initial purchase of the device) and an additional cost of replacing the device every five years was applied as long as the given strategy called for continued wearable screening.

For all screening strategies, a one-time nurse visit cost was incurred upon screening. Also, for strategies involving a wrist-worn wearable followed by a confirmatory patch monitor, an additional nursing visit cost was incurred after an abnormal wearable signal for prescription and application of the patch monitor.

Lastly, a physician visit cost was incurred for all instances where an ultimate diagnosis of AF was made (either true or false positive), corresponding to diagnosis counseling and prescription of anticoagulation if appropriate (i.e., no history of major bleeding).

© 2022 Chen W et al. *JAMA Health Forum.*

Modeling of paroxysmal AF

Given lack of reliable data regarding the test characteristics of wearable devices for detecting paroxysmal AF over longer durations of monitoring (i.e., months to years), we modeled the temporal effect of screening via a wearable device as follows:

We applied literature-based values for the estimated prevalence of paroxysmal AF among individuals with screen-detected AF (59%).[1–4] We then utilized estimates of the average AF burden among individuals with paroxysmal AF (4.5%).[4–6] We assumed that the average AF burden follows a uniform distribution on the order of days (i.e., an individual with an AF burden of 4.5% would be expected, on average, to spend 4.5% of each day in AF).

Then, the probability that an individual will not experience a single AF episode over *t* days is $(1\text{-}0.045)^t$. The probability that an individual will experience at least one AF episode over *t* days is the complement, or $1\text{-}(1\text{-}0.045)^t$. We then applied the known static test characteristics of the wearable device to the probability of observing AF with each cycle of simulation (i.e., one month or 30 days).

For example, an individual with AF wearing a watch for 3 months would have a probability of the device being exposed to an AF episode after one cycle of $1\text{-}(1\text{-}0.045)^{30}$, or 0.749. If this individual is wearing a W-PPG (sensitivity 95.3, specificity 99.7), they will be diagnosed with AF with probability 0.749 * 0.953, or 0.714 after one cycle. As with other screening modalities, if a diagnosis of AF is not made, and the screening strategy under evaluation includes continued screening, then the screening process will repeat as dictated by the length of the screening interval being evaluated. In this case of 3-month screening, screening would continue for three cycles, with a probability of being diagnosed with AF of 0.714 after each cycle, and the overall probability of being diagnosed with AF of $1\text{-}(1\text{-}0.714)^3$ or 0.977.

Although the data provided by a recent study by Diedrichsen et al. are insufficient to primarily inform test characteristics over the necessary durations required to model wearable screening approaches, we were able to validate that our approach described above resulted in comparable estimates of sensitivity for paroxysmal AF at 30 days, after allowance for the uncertainty in AF burden, which we modeled in probabilistic sensitivity analyses (**Table A**).[7]

eMethods Table A. Probability of AF episode with 30 days of monitoring

Method	AF burden value	Probability
AF model (lower bound)	0.011	0.282
AF model (base)	0.045	0.749
AF model (upper bound)	0.17	0.996
Diedrichsen et al.[7]	-	0.34

© 2022 Chen W et al. *JAMA Health Forum.*

Sensitivity analysis assumptions

In cases where uncertainty in model parameters could not be estimated based on the available published literature, we varied point estimates by +/- 20% when performing both one-way and probabilistic sensitivity analyses.

Simulation size determination

To determine sufficient cohort size for base case simulation taking into account first-order uncertainty (i.e., Monte Carlo error), we followed the guidelines provided by the ISPOR-SMDM Modeling Good Research Practices Task Force Working Group-6.[8] Specifically, we tested results at increasing sample size from 10 million to 50 million and noted the comparative clinical effectiveness of all 8 screening strategies with respect to no screening, i.e., d(QALY), as well as the cost effectiveness results for all 5 cases. We report these values in the tables below. At a precision of 0.001 (i.e., 100 QALYs per 100,000 persons), one can see that d(QALY) is well-stabilized at simulation sizes at or above 30 million (**Table B**). Further, the cost-effectiveness strategy remained the same for all simulation sizes and the ICER stabilizes at a precision of $100,000 at or above 30 million (**Table C**). As a result, we utilized a simulation size of 30 million for the base case analysis.

eMethods Table B. Comparative clinical effectiveness across various simulation size

#							d(QALY) at a given simulation cohort size (million)				
Rank	PP	12L	PPG	1L	PM	Freq	50	40	30	20	10
1			X	X	X	life	0.00933	0.00961	0.00957	0.00907	0.01068
2	X	X	X	X	X	life	0.00854	0.00892	0.00866	0.00869	0.01068
3		X	X	X	X	life	0.00562	0.00586	0.00596	0.00591	0.00720
4			X		X	life	0.00536	0.00551	0.00561	0.00531	0.00632
5	X	X	X		X	life	0.00490	0.00486	0.00486	0.00405	0.00630
6		X	X		X	life	0.00220	0.00253	0.00226	0.00161	0.00203
7	X	X				once	0.00049	0.00077	0.00093	0.00027	0.00089
8		X				once	-0.0016	-0.0013	-0.0012	-0.0013	-0.0020

eMethods Table C. Cost-effectiveness results across various simulation size

PP	12L	PPG	1L	PM	Freq	QALY	cost	ICER
50 million								
X	X				once	7.09192	30174	Reference
		X	X	X	life	7.10076	30666	55622
40 million								
X	X				once	7.09220	30167	Reference
		X	X	X	life	7.10104	30670	56833
30 million								
X	X				once	7.09249	30169	Reference
		X	X	X	life	7.10113	30669	57882
20 million								

© 2022 Chen W et al. *JAMA Health Forum.*

X	X				once	7.09257	30180	Reference
		X	X	X	life	7.10137	30675	56273
10 million								
X	X				once	7.09159	30192	Reference
		X	X	X	life	7.10138	30665	48325

© 2022 Chen W et al. *JAMA Health Forum.*

eTable 1. Disease Incidence (per 1000 Person-Years)

Incidence of clinically recognized AF						
	< 55 years	55 to 64 years	65 to 74 years	75 to 84 years	≥ 85 years	References
**Male**	0.62 (0.62-0.76)	4.34 (4.31-4.56)	12.91 (9.24-14.33)	24.52 (19.80-26.31)	39.66 (15.6[9]-46.81)	9
**Female**	0.19 (0.19-0.21)	2.16 (1.10-3.70[9])	6.79 (5.91-7.65[9])	17.14 (14.40[9]-17.69)	27.69 (11.9[9]-28.67)	

Incidence of stroke (for no AF and no treatment group)								
	< 35 years	35 to 44 years	45 to 54 years	55 to 64 years	65 to 74 years	75 to 84 years	≥ 85 years	References
**Male**	0.03 (0-0.19)	0.27 (0.07-0.81)	0.73 (0.33-1.38)	1.77 (1.03-2.84)	6.46 (4.70-8.68)	9.42 (6.56-13.10)	19.72 (11.49-31.58)	10
**Female**	0.06 (0-0.25)	0.16 (0.02-0.57)	0.54 (0.05-1.17)	1.75 (1.00-2.84)	4.08 (2.71-5.89)	10.51 (7.89-13.71)	15.08 (10.17-21.52)	

Incidence of stroke (for AF and no treatment group)				
**CHA_2DS_2-VASc Score**	**Base**	**Lower**	**Upper**	**References**
0	2			11
1	6			
2	25			
3	37			
4	55			
5	84			
6	114			
7	131			
8	126			
9	144			

Incidence of intracranial hemorrhage				
	Base	Lower	Upper	References
**No treatment (converted from probability at 7.4y)**	0.81			12
**Aspirin**	0.95	0.95[12]	4	12,13
**Warfarin**	7.8 (WA)	3.3[14]	8.5[15]	14–17
**DOAC**	3.99 (WA)	3.3[14]	5.0[17]	14–17

© 2022 Chen W et al. *JAMA Health Forum.*

**OAC+aspirin**	16.0			[18]

**Incidence of major hemorrhage**				
	**Base**	**Lower**	**Upper**	**References**
**No treatment**	1.64[19]	0.467	1.64	[19,20]
**Aspirin**	2.31[19]	1.92[19]	8.0[21]	[19,21]
**Warfarin**	31.2 (WA)	16.9[14]	34.3[15]	[14–17]
**DOAC**	29.0 (WA)	9.6[14]	36.0[17]	[14–17]
**OAC+aspirin**	43.0			[18]

**Incidence of clinically relevant non-major hemorrhage**				
	**Base**	**Lower**	**Upper**	**References**
**No treatment**	2.9 (A)	2.2[22]	3.6[23]	[22,23]
**Aspirin (converted from probability at 2.3y)**	5.61			[24]
**Warfarin**	107.1 (WA)	101.5[15]	114.0[17]	[15,17]
**DOAC**	102.2 (WA)	86.7	118.0	[15,17]
**OAC+aspirin (HR versus warfarin)**	1.19	0.36	4.17	[18]

© 2022 Chen W et al. *JAMA Health Forum.*

eTable 2. Comorbidity Prevalence/Incidence (per 1000 Person-Years)

Incidence of heart failure							
	**55-64 years**	**65-69 years**	**70-74 years**	**75-79 years**	**80-84 years**	**≥85 years**	**References**
**Male**	3.9 (3.9-11.2)[25]	7.4 (6.4-8.5)	10.8 (9.2-12.5)	16.9 (14.3-19.5)	29.4 (24.1-34.8)	45.6 (35.3-55.8)	[25,26]
**Female**	2.7 (2.7-8.2)[25]	5.1 (4.3-5.9)	10.2 (8.8-11.6)	14.4 (12.3-16.5)	23.2 (19.5-26.8)	41.1 (34.8-47.4)	
Defined using presence of Framingham heart failure criteria[27]							

Prevalence of heart failure					
	**20-39 years**	**40-59 years**	**60-79 years**	**≥80 years**	**References**
**Male**	0.3	1.2	6.9	12.8	[25]
**Female**	0.2	1.7	4.8	12.0	
Defined using NHANES 2013-2016 health interviews. Heart failure was considered present if a person reported "yes" to being told by a healthcare professional that he or she had heart failure.					

Incidence of hypertension							
	**20-29 years**	**30-39 years**	**40-49 years**	**50-59 years**	**60-69 years**	**70-79 years**	**References**
**Male**	8.15 (5.5-10.0)	16.6 (1.3-75.0)	21.9 (3.9-71.0)	23.6 (8.7-91.0)	28.0 (10.2-88.6)	31.1	[28]
**Female**	3.3 (2.0-4.6)	7.7 (6.8-33.0)	18.0 (16.1-57.0)	24.9 (32.4-66.0)	34.7 (42.6-95.8)	42.8	
Defined using systolic blood pressure ≥ 160mmHg or diastolic blood pressure ≥ 95mmHg on two consecutive measurements, or use of anti-hypertensive medication							

Prevalence of hypertension							
	**20-34 years**	**35-44 years**	**45-54 years**	**55-64 years**	**65-74 years**	**≥75 years**	**References**
**Male**	25.7	42.5	56.3	66.4	70.8	80.0	[25]
**Female**	13.0	31.6	49.7	64.9	77.8	85.6	
Defined using NHANES 2013-2016 blood pressure measurements and health interviews. Hypertension was considered present if a person had systolic blood pressure ≥ 130mmHg or diastolic blood pressure ≥ 80mmHg, reported "yes" to taking anti-hypertensive medication, or reported "yes" to being told by a healthcare professional that he or she had hypertension on at least two occasions.							

Incidence of diabetes		
	**≥20 years**	**References**

© 2022 Chen W et al. *JAMA Health Forum.*

**Male**	4.15 (4.15-6.15)	[29]
**Female**	2.70 (2.70-6.79)	
Defined as fasting glucose ≥ 126 mg/dL, 2-hour post-challenge glucose ≥ 200 mg/dL, random glucose ≥ 200 mg/dL with presence of hyperglycemia symptoms, hemoglobin a1c ≥ 6.5%		

**Prevalence of diabetes**		
	**≥20 years**	**References**
**Male**	15.5	[25]
**Female**	11.7	
Defined as fasting glucose ≥ 126 mg/dL, 2-hour post-challenge glucose ≥ 200 mg/dL, hemoglobin a1c ≥ 6.5%, or use of anti-glycemic medications		

**Incidence of myocardial infarction (MI)**						
	**35-44 years**	**45-54 years**	**55-64 years**	**65-74 years**	**≥ 75 years**	**References**
**Male**	0.79 (0.79-2.35)	2.14 (2.14-4.01)	3.82 (3.82-7.05)	7.26 (7.26-10.67)	9.39 (9.39-15.9)	[25]
**Female**	0.27 (0.27-1.05)	0.99 (0.99-2.70)	2.10 (2.10-4.35)	3.69 (3.69-7.70)	8.53 (8.53-12.0)	
Defined using the Atherosclerosis Risk in Communities Study acute myocardial infarction surveillance definition[30]						

**Prevalence of MI**					
	**20-39 years**	**40-59 years**	**60-79 years**	**≥ 80 years**	**References**
**Male**	0.1	2.8	11.5	17.3	[25]
**Female**	0.4	2.1	4.2	12.7	
Defined using NHANES 2013-2016 health interviews. Myocardial infarction was considered present if a person reported "yes" to being told by a healthcare professional that he or she ever had a heart attack or myocardial infarction.					

**Incidence of peripheral arterial disease (PAD)**					
	**50-59 years**	**60-69 years**	**70-79 years**	**≥ 80 years**	**References**
**Overall**	1.0	2.0	2.8	3.5	[31]
**Female (vs. Male)**	Relative risk/incidence ratio: 0.538				
Defined using presence of Read diagnosis codes indicative of a symptomatic PAD diagnosis or related revascularization procedures					

© 2022 Chen W et al. *JAMA Health Forum.*

Prevalence of PAD						
	**40-49 years**	**50-59 years**	**60-69 years**	**70-79 years**	**≥ 80 years**	**References**
**Male**	1.4 (0.2-2.6)	1.9 (0.9-5.0)	5.4 (3.5-13.2)	9.2 (9.2-24.4)	22.6 (21.5-59.0)	[25]
**Female**	1.9 (0-3.0)	4.3 (0.4-4.3)	5.1 (0.7-8.9)	7.9 (6.9-20.0)	18.2 (18.2-35.1)	
Defined using ankle-brachial index < 0.9 or previous revascularization for PAD						

Incidence of coronary disease (including both MI and non-MI CAD)				
	**35-54 years**	**55-69 years**	**≥70 years**	**References**
**Male**	2.06	6.33	15.5	[32]
**Female**	0.57	2.82	9.52	
Defined using International Classification of Diseases, 9th revision (ICD-9) and ICD, 10th revision (ICD-10) codes: 410-414, I21-I25 applied to hospital admission data and cause of death register				

Prevalence of coronary disease					
	**20-39 years**	**40-59 years**	**60-79 years**	**≥ 80 years**	**References**
**Male**	0.5	6.1	19.7	31.0	[25]
**Female**	1.0	6.2	12.6	25.4	
Defined using NHANES 2013-2016 health interviews. Coronary heart disease was considered present if a person reported “yes” to being told by a healthcare professional that he or she had coronary heart disease, angina or angina pectoris, heart attack, or myocardial infarction. Those who answered “no” but were diagnosed with angina based on the Rose questionnaire were also included.					

Conditional prevalence		
**Condition**	**Value**	**References**
Prevalence (PAD | non-MI CAD)	0.141	[33]
Prevalence (PAD | MI)	0.048	[34–36]
Prevalence (PAD | no CAD)	0.0090	
Prevalence (non-MI CAD | PAD)	0.109	[38]
Prevalence (MI | PAD)	0.182	[38]

© 2022 Chen W et al. *JAMA Health Forum.*

eTable 3. Disease Recurrence Rates (Monthly Probabilities)

	Base	Lower	Upper	References
Ischemic stroke				
No treatment				
First year	0.0115	0.00874	0.0144	[39]
Subsequent years	0.00348	0.00141	0.00668	[39]
Aspirin				
First year	0.009			Use RR
Subsequent years	0.003			Use RR
OAC (with or without aspirin)				
First year	0.004			Use RR
Subsequent years	0.001			Use RR
Intracranial hemorrhage				
First year	0.0135			[40]
Subsequent years	Baseline incidence			

© 2022 Chen W et al. *JAMA Health Forum.*

eTable 4. Disease-Related Mortality (Monthly Probabilities)

	Base	Lower	Upper	References
Ischemic stroke (30-day, AF)				
Mild	0.01			[41]
Moderate	0.13			[41]
Severe	0.39			[41]
Ischemic stroke (first year among 30-day survivors, no AF)				
Mild	0			Assumption
Moderate-severe	Use RR			
Ischemic stroke (first year among 30-day survivors, AF)				
Mild	0			Assumption
Moderate-severe	0.026			[42]
Ischemic stroke (subsequent years among 1-year survivors, no AF)				
	**Base**	**Lower**	**Upper**	**References**
Mild	0			Assumption
Moderate-severe	Use RR			
Ischemic stroke (subsequent years among 1-year survivors, AF)				
Mild	0			Assumption
Moderate-severe	0.0077			[42]
**Relative risk of ischemic stroke mortality (AF versus no AF)**	1.63	1.25	2.00	[42,43]
Intracranial hemorrhage (disabling)				
30-day probability of death (aspirin or no treatment)	0.35	0.332	0.374	[44]
Odds ratio for death at 30 days (OAC or OAC+aspirin)	3	1.9	4.7	[45]
First and Subsequent years among 30-day survivors	0.01575			[46]
Major hemorrhage				
No treatment	0.091			[47]
Aspirin	0.078			[47]
Warfarin	0.14	0.112	0.206	[48]

© 2022 Chen W et al. *JAMA Health Forum.*

DOAC	0.082	0.068	0.104	[14,15,17]
OAC+Aspirin	0.11			Assumption

© 2022 Chen W et al. *JAMA Health Forum.*

eTable 5. Severity Measures

	Base	Lower	Upper	References
Ischemic Stroke				
No AF, No Treatment				
Proportion of ischemic strokes that are mild (mRS 0-2)	0.47	0.375	0.575	[49]
Proportion of ischemic strokes that are moderate (mRS 3-4)	0.405	0.3	0.5	[49]
Proportion of ischemic strokes that are severe or fatal (mRS 5-6)	0.125	0.07	0.16	[49]
AF, No Treatment				
	**Base**	**Lower**	**Upper**	**References**
Proportion of ischemic strokes that are mild (mRS 0-2)	0.363	0.3	0.45	[49]
Proportion of ischemic strokes that are moderate (mRS 3-4)	0.364			[49]
Proportion of ischemic strokes that are severe or fatal (mRS 5-6)	0.273			[49]
AF, on OAC				
Proportion of ischemic strokes that are mild (mRS 0-2)	0.47			[41]
Proportion of ischemic strokes that are moderate (mRS 3-4)	0.42			[41]
Proportion of ischemic strokes that are severe or fatal (mRS 5-6)	0.11			[41]
Intracranial hemorrhage				
Proportion of intracranial hemorrhages that are nondisabling	0.26	0.12	0.39	[44]

© 2022 Chen W et al. *JAMA Health Forum.*

eTable 6. Utilities

	Base	Lower	Upper	References
Atrial Fibrillation				
Asymptomatic*	0.954			[50]
Symptomatic	0.81	0.68	0.91	[51]
Ischemic stroke				
	**Base**	**Lower**	**Upper**	**References**
Mild stroke (mRS 0-2)	0.89	0.80	0.93	[49]
Moderate stroke (mRS 3-4, first year)	0.67	0.56	0.71	[49]
Moderate stroke (mRS 3-4, subsequent years)	0.71	0.67	0.80	[49]
Severe or fatal stroke (mRS 5-6, first year)	0.30	0.20	0.40	[49]
Severe stroke (mRS 5, subsequent years)	0.48	0.30	0.60	[49]
Intracranial hemorrhage				
Nondisabling	0.89			[49]
Disabling (first year)	0.42			[49]
Disabling (subsequent years)	0.55			[49]
Major bleeding				
1 month	0.8			[49]
Therapeutics (while receiving)				
Warfarin	0.987	0.953	1.0	[52]
Novel oral anticoagulants	0.994	0.993	0.996	[53]
Aspirin	0.998	0.994	1	[52]
*Proportion of AF that is asymptomatic estimated to be 12%[54]				

© 2022 Chen W et al. *JAMA Health Forum.*

eTable 7. Costs

Treatment-related cost			
	Base	Range	Reference
Warfarin drug cost (monthly)	15.4	10.4-68.4	[55]
Warfarin INR testing (monthly)	7.9	2.6-15.8	[55]
MD visit (annual)	76.2	52.3-83.2	
NOAC			
NOAC Drug cost (monthly)	289.0	148.6-399.5	[55]
OAC Drug cost (monthly)	Weighted average of Warfarin and NOAC drug cost		
OAC INR testing (monthly)	Warfarin INR testing cost scaled by proportion taking warfarin		
MD visit (annual)	76.2	52.3-83.2	[56]
Ischemic stroke (IS)-related cost			
Upfront (first month)			
Mild IS (mRS 0-2)	11917	10712-15000	[57]
Moderate IS (mRS 3-4)	17885	15009-19120	[57]
Severe IS (mRS 5-6)	22648	19442-32360	[57]
Maintenance (starting from 2nd month, monthly)			
Mild IS (mRS 0-2)	650	570-729	[58]
Moderate IS (mRS 3-4)	2355	1247-3463	[58]
Severe IS (mRS 5-6)	4824	2355-7292	[58]
Intracranial hemorrhage (ICH)-related cost			
Upfront (first month)			
Non-disabling ICH	24961	16646-37163	[57]
Disabling ICH	78897	53526,116485	[57]
Maintenance (starting from 2nd month, monthly)			
Non-disabling ICH	1746	1397-2095 (+/20%)	[58]
Disabling ICH	3127	2502-3752 (+/10%)	[58]
Major bleed-related cost			
Upfront (first month)	11801	6703,45612	[57]

© 2022 Chen W et al. *JAMA Health Forum.*

Minor bleed-related cost			
Upfront (first month)	148.3	113.7-162.1	[56]
Screening-related cost			
PP	0.78	0.31-0.85	[56]
1-lead ECG	14.7	12.5-14.7	[59]
12-lead ECG	17.3	17.3-32	[56]
PM	159	159-199	[60]
watch	232.5		
MD visit (at confirmatory)	76.2	52.3-83.2	[56]
RN visit (at initial and transition from watch to PM)	23.5	9.4-25.6	[56]
INR = International Normalized Ratio; IS = ischemic stroke; ICH = intracranial hemorrhage; NOAC = novel oral anticoagulant; OAC = oral anticoagulant; mRS = modified Rankin score. All costs have been adjusted to 2020 US dollars.[61,62]			

© 2022 Chen W et al. *JAMA Health Forum.*

eTable 8. Summary of Parameters Included in Sensitivity Analyses

Parameter	Included in one-way sensitivity analysis	Included in probability sensitivity analysis (PSA)	Distribution(s) utilized in PSA
**Incidence rates**			
Atrial fibrillation		X	Log-normal, beta
Ischemic stroke (AF)	X	X	Log-normal, beta
Ischemic stroke (non-AF)		X	Log-normal, beta
Intracranial hemorrhage	X	X	Log-normal, beta
Major hemorrhage	X	X	Log-normal, beta
Recurrent stroke		X	Log-normal, beta
Mortality			
Ischemic stroke	X	X	Beta
Intracranial hemorrhage	X	X	Beta
Major hemorrhage		X	Beta
**Severity**			
Ischemic stroke		X	Beta
Intracranial hemorrhage		X	Beta
**Other clinical factors**			
Proportion of AF that is undiagnosed	X	X	Beta
Proportion of AF that is persistent	X	X	Beta
Average AF burden in paroxysmal AF	X	X	Beta
Proportion of OAC that is DOAC	X	X	Beta
OAC discontinuation rate	X	X	Beta
Patch monitor adherence	X	X	Triangular
Effect of OAC on ischemic stroke	X	X	Beta
**Test characteristics**			
Pulse palpation	X	X	Beta
Single-lead ECG	X	X	Beta
Patch monitor	X	X	Beta, Triangular
12-lead ECG	X	X	Beta
Smart watch/band PPG	X	X	Beta
Smart watch/band ECG	X	X	Beta
**Utilities**			
AF	X	X	Beta
Ischemic stroke		X	Beta
OAC		X	Beta
Aspirin		X	Beta
**Costs**			
Drug-related cost	X	X	Gamma
MD visit cost	X	X	Gamma
RN visit cost	X	X	Gamma
Ischemic stroke-related cost	X	X	Gamma
Intracranial hemorrhage-related cost	X	X	Gamma
Major bleed-related cost	X	X	Gamma
Pulse palpation cost	X	X	Gamma
Single-lead ECG cost	X	X	Triangular
12-lead ECG cost	X	X	Gamma
Patch monitor cost	X	X	Triangular

© 2022 Chen W et al. *JAMA Health Forum.*

eTable 9. Summary of Costs Associated With Each Screening Strategy

Strategy						Screening Cost ($)	Treatment Cost ($)	Bleeding-related Cost ($)	Stroke-related Cost ($)	Overall Cost ($)
PP	12L	PPG	1L	PM	freq					
X	X				Once	45.00	1,197.90	2,748.80	26,190.60	30,182.30
		X	X	X	Life	614.40	1,216.10	2,807.00	26,044.90	30,682.50
No screening						19.20	1,131.80	2,702.90	26,370.90	30,224.80
	X				Once	57.80	1,272.60	2,794.60	26,160.60	30,285.70
	X	X		X	Life	583.30	1,350.50	2,886.00	26,007.70	30,827.50
X	X	X		X	Life	578.70	1,281.90	2,841.20	26,033.20	30,735.00
		X		X	Life	581.20	1,257.30	2,856.60	26,034.80	30,729.90
	X	X	X	X	Life	616.00	1,310.80	2,836.60	26,008.80	30,772.20
X	X	X	X	X	Life	611.90	1,240.40	2,800.50	26,045.40	30,698.20
Costs presented per simulated individual										

© 2022 Chen W et al. *JAMA Health Forum.*

eTable 10. True and false Positive Rates by Strategy

Strategy						Total number of individuals with AF (millions)	True AF cases detected (millions)	False AF diagnoses made (millions)	Total AF diagnoses made (millions)	AF incidence rate (per 1,000 person-yr)	AF true positive rate (%)	AF false positive rate (%)*
PP	12L	PPG	1L	PM	Freq							
		X	X	X	life	10.760	8.768	0.076	8.844	25.592	81.5	0.4
X	X	X	X	X	life	10.767	8.793	0.139	8.932	25.588	81.7	0.7
	X	X	X	X	life	10.769	8.831	0.405	9.237	25.581	82.0	2.1
		X		X	life	10.779	8.815	0.275	9.091	25.574	81.8	1.4
X	X	X		X	life	10.790	8.842	0.337	9.180	25.571	81.9	1.8
	X	X		X	life	10.792	8.879	0.601	9.479	25.570	82.3	3.1
X	X				once	10.796	8.533	0.135	8.668	25.593	79.0	0.7
No screening						10.797	8.230	0.072	8.372	25.597	76.9	0.4
	X				once	10.793	8.604	0.401	9.006	25.598	79.7	2.1

*False positive rate in no screening condition attributable to application of patch monitor following stroke events

© 2022 Chen W et al. *JAMA Health Forum.*

eTable 11. Cost-effectiveness Results for Scenario Analyses

PP	12L	PPG	1L	PM	freq	Quality-adjusted life-years (QALY)	Cost ($)	Incremental Cost-effectiveness Ratio ($/QALY)
Cost of wrist-worn wearable = $150								
X	X				Once	7.09249	30,182	Reference
		X	X	X	Life	7.10113	30,481	34,583
No screening						7.09156	30,225	Strongly dominated
	X				Once	7.09040	30,286	Strongly dominated
	X	X		X	Life	7.09382	30,656	Strongly dominated
X	X	X		X	Life	7.09642	30,561	Strongly dominated
		X		X	Life	7.09717	30,555	Strongly dominated
	X	X	X	X	Life	7.09752	30,575	Strongly dominated
X	X	X	X	X	Life	7.10022	30,499	Strongly dominated
Cost of wrist-worn wearable = $200								
X	X				Once	7.09249	30,182	Reference
		X	X	X	Life	7.10113	30,603	48,704
No screening						7.09156	30,225	Strongly dominated
	X				Once	7.09040	30,286	Strongly dominated
	X	X		X	Life	7.09382	30,760	Strongly dominated
X	X	X		X	Life	7.09642	30,657	Strongly dominated
		X		X	Life	7.09717	30,661	Strongly dominated
	X	X	X	X	Life	7.09752	30,695	Strongly dominated
X	X	X	X	X	Life	7.10022	30,620	Strongly dominated
Cost of wrist-worn wearable = $250								
X	X				Once	7.09249	30,182	Reference
		X	X	X	Life	7.10113	30,725	62,836
No screening						7.09156	30,225	Strongly dominated
	X				Once	7.09040	30,286	Strongly dominated
	X	X		X	Life	7.09382	30,864	Strongly dominated
X	X	X		X	Life	7.09642	30,772	Strongly dominated
		X		X	Life	7.09717	30,767	Strongly dominated
	X	X	X	X	Life	7.09752	30,814	Strongly dominated
X	X	X	X	X	Life	7.10022	30,741	Strongly dominated
Cost of wrist-worn wearable = $300								
X	X				Once	7.09249	30,182	Reference
		X	X	X	Life	7.10113	30,847	76,956
No screening						7.09156	30225	Strongly dominated

© 2022 Chen W et al. *JAMA Health Forum.*

	X				Once	7.09040	30,286	Strongly dominated
	X	X		X	Life	7.09382	30,968	Strongly dominated
X	X	X		X	Life	7.09642	30,877	Strongly dominated
		X		X	Life	7.09717	30,873	Strongly dominated
	X	X	X	X	Life	7.09752	30,933	Strongly dominated
X	X	X	X	X	Life	7.10022	30,862	Strongly dominated
Daily wear time of wrist-worn wearable = 6 hours								
X	X				Once	7.09249	30,182	Reference
		X	X	X	Life	7.10128	30,677	56,314
No screening						7.09156	30,225	Strongly dominated
	X				Once	7.09040	30,286	Strongly dominated
	X	X		X	Life	7.09363	30,825	Strongly dominated
X	X	X		X	Life	7.09596	30,733	Strongly dominated
		X		X	Life	7.09714	30,735	Strongly dominated
	X	X	X	X	Life	7.09739	30,765	Strongly dominated
X	X	X	X	X	Life	7.10033	30,697	Strongly dominated
Daily wear time of wrist-worn wearable = 12 hours								
X	X				Once	7.09249	30,182	Reference
		X	X	X	Life	7.10106	30,678	57,795
No screening						7.09156	30,225	Strongly dominated
	X				Once	7.09040	30,286	Strongly dominated
	X	X		X	Life	7.09376	30,826	Strongly dominated
X	X	X		X	Life	7.09632	30,732	Strongly dominated
		X		X	Life	7.09739	30,732	Strongly dominated
	X	X	X	X	Life	7.09769	30,769	Strongly dominated
X	X	X	X	X	Life	7.10066	30,702	Strongly dominated
Daily wear time of wrist-worn wearable = 24 hours								
X	X				Once	7.09249	30,182	Reference
		X	X	X	Life	7.10092	30,682	59,288
No screening						7.09156	30,225	Strongly dominated
	X				Once	7.09040	30,286	Strongly dominated
	X	X		X	Life	7.09386	30,826	Strongly dominated
X	X	X		X	Life	7.09652	30,735	Strongly dominated
		X		X	Life	7.09725	30,729	Strongly dominated
	X	X	X	X	Life	7.09759	30,776	Strongly dominated
X	X	X	X	X	Life	7.10039	30,697	Strongly dominated
Men								
X	X				Once	6.83680	30,778	Reference
		X	X	X	Life	6.84374	31,197	60,375

© 2022 Chen W et al. *JAMA Health Forum.*

No screening						6.83538	30,829	Strongly dominated
	X				Once	6.83545	30,864	Strongly dominated
	X	X		X	Life	6.83793	31,344	Strongly dominated
X	X	X		X	Life	6.83977	31,250	Strongly dominated
		X		X	Life	6.83911	31,236	Strongly dominated
	X	X	X	X	Life	6.84108	31,282	Strongly dominated
X	X	X	X	X	Life	6.84333	31,201	Strongly dominated
Women								
X	X				Once	7.34832	29,586	Reference
		X	X	X	Life	7.35847	30,168	57,340
No screening						7.34786	29,621	Strongly dominated
	X				Once	7.34547	29,708	Strongly dominated
	X	X		X	Life	7.34980	30,311	Strongly dominated
X	X	X		X	Life	7.35321	30,219	Strongly dominated
		X		X	Life	7.35521	30,224	Strongly dominated
	X	X	X	X	Life	7.35406	30,263	Strongly dominated
X	X	X	X	X	Life	7.35722	30,196	Strongly dominated
Specificity of W-ECG = 80%								
X	X				Once	7.09249	30,182	Reference
		X	X	X	Life	7.09988	30,698	69,891
No screening						7.09156	30,225	Strongly dominated
	X				Once	7.09040	30,286	Strongly dominated
	X	X		X	Life	7.09382	30,828	Strongly dominated
X	X	X		X	Life	7.09642	30,735	Strongly dominated
		X		X	Life	7.09717	30,730	Strongly dominated
	X	X	X	X	Life	7.09700	30,788	Strongly dominated
X	X	X	X	X	Life	7.09977	30,699	Strongly dominated
Specificity of W-ECG = 85%								
X	X				Once	7.09249	30,182	Reference
		X	X	X	Life	7.10045	30,691	63,945
No screening						7.09156	30,225	Strongly dominated
	X				Once	7.09040	30,286	Strongly dominated
	X	X		X	Life	7.09382	30,828	Strongly dominated
X	X	X		X	Life	7.09642	30,735	Strongly dominated
		X		X	Life	7.09717	30,730	Strongly dominated
	X	X	X	X	Life	7.09730	30,780	Strongly dominated
X	X	X	X	X	Life	7.09956	30,699	Strongly dominated
Specificity of W-PPG = 80%								
X	X				Once	7.09249	30,182	Reference

© 2022 Chen W et al. *JAMA Health Forum.*

		X	X	X	Life	7.10029	30,706	67,203
No screening						7.09156	30,225	Strongly dominated
	X				Once	7.09040	30,286	Strongly dominated
	X	X		X	Life	7.08290	30,915	Strongly dominated
X	X	X		X	Life	7.08525	30,823	Strongly dominated
		X		X	Life	7.08291	30,861	Strongly dominated
	X	X	X	X	Life	7.09677	30,776	Strongly dominated
X	X	X	X	X	Life	7.09904	30,703	Strongly dominated
Specificity of W-PPG = 85%								
X	X				Once	7.09249	30,182	Reference
		X	X	X	Life	7.10008	30,710	69,489
No screening						7.09156	30,225	Strongly dominated
	X				Once	7.09040	30,286	Strongly dominated
	X	X		X	Life	7.08331	30,908	Strongly dominated
X	X	X		X	Life	7.08583	30,823	Strongly dominated
		X		X	Life	7.08297	30,877	Strongly dominated
	X	X	X	X	Life	7.09695	30,765	Strongly dominated
X	X	X	X	X	Life	7.09903	30,694	Strongly dominated
Sensitivity of W-PPG = 80%								
X	X				Once	7.09249	30,182	Reference
		X	X	X	Life	7.10115	30,683	57,874
No screening						7.09156	30,225	Strongly dominated
	X				Once	7.09040	30,286	Strongly dominated
	X	X		X	Life	7.09383	30,827	Strongly dominated
X	X	X		X	Life	7.09632	30,734	Strongly dominated
		X		X	Life	7.09738	30,737	Strongly dominated
	X	X	X	X	Life	7.09767	30,770	Strongly dominated
X	X	X	X	X	Life	7.10059	30,697	Strongly dominated
Sensitivity of W-PPG = 85%								
X	X				Once	7.09249	30,182	Reference
		X	X	X	Life	7.1011	30,687	58,602
No screening						7.09156	30,225	Strongly dominated
	X				Once	7.09040	30,286	Strongly dominated
	X	X		X	Life	7.09379	30,829	Strongly dominated
X	X	X		X	Life	7.09634	30,734	Strongly dominated
		X		X	Life	7.09736	30,736	Strongly dominated
	X	X	X	X	Life	7.09772	30,774	Strongly dominated
X	X	X	X	X	Life	7.10046	30,700	Strongly dominated

© 2022 Chen W et al. *JAMA Health Forum.*

eTable 12. One-Way Sensitivity Analysis

			Strategy					
			**No screening**	**1**	**2**	**3**	**4**	**5**
	**Freq**			once	life	life	once	life
	**PP**			X		X		
	**12L**			X		X	X	
	**PPG**				X	X		X
	**1L**				X	X		
	**PM**				X	X		X
**Parameter**	**Value**		**ICER**					
baseline diagnosis rate	0.67			*	52809			
	0.73			*	57208			
proportion of AF that is persistent	0.04			*	53815			
	0.66			*	55392			
paroxysmal AF burden	0.011			*	58523			
	0.17			*	57796			
proportion of OAC that is NOAC (vs. Warfarin)	0.10			*	55253			
	0.50			*	61474			
probability of Warfarin discontinuation (monthly)	0.007			*	59767			
	0.042			*	71576	57270		
RR of NOAC discontinuation (vs. Warfarin)	0.57			*	56314			
	0.84			*	58784			
utility of symptomatic AF	0.68			*	49770			
	0.91			*	66075			
RR of ischemic stroke for AF patients (OAC vs. placebo)	0.23			*	50594			
	0.46			*	56193			
RR of ischemic stroke for non-AF patients (OAC vs. placebo)	0.44			*	58247			
	0.76			*	56950			

© 2022 Chen W et al. *JAMA Health Forum.*

Uptake of patch monitor	0.62			*	59904			
major bleeding incidence rate with Warfarin	16.9			*	54938			
	34.3			*	55721			
major bleeding incidence rate with NOAC	9.6			*	61361			
	36.0			*	56563			
ICH incidence rate with Warfarin	3.3			*	58086			
	8.5			*	57438			
ICH incidence rate with NOAC	3.3			*	57807			
	5.0			*	57722			
severe ischemic stroke 30-day all-cause mortality	0.312			*	54415			
	0.468			*	58718			
OR of 30-day all-cause mortality for ICH (OAC vs. placebo)	1.9			*	57396			
	4.7			*	57861			
12L ECG sensitivity	81.0			*	56968			
	100.0			*	59107			
12L ECG specificity	76.0		*		47822			
	100.0			*	65428		18911	
watch PPG sensitivity	92.0			*	58227			
	97.4			*	57506			
watch PPG specificity	89.7			*	65890			
	100.0			*				56610
watch ECG sensitivity	76.7			*	57617			
	98.3			*	59815			
watch ECG specificity	89.6			*	59112			
	100.0			*	58038			
patch monitor sensitivity	90.0			*	59306			
patch monitor specificity	86.9			*	59798			
	100.0			*	57450			
	16.0			*	50504			

© 2022 Chen W et al. *JAMA Health Forum.*

pulse palpation sensitivity	100.0			*	59893			
pulse palpation specificity	65.0			*	49937			
	91.0			*		57383		
RR of ischemic stroke (paroxysmal vs. persistent AF)	0.01			*				
	1.00			*	50746			
RR of ischemic stroke for AF patients (aspirin vs. placebo)	0.65			*	56637			
	0.94			*	56017			
Warfarin monthly drug cost	10.4			*	57817			
	68.4			*	58687			
INR testing cost	2.6			*	57813			
	15.8			*	58010			
MD visit cost	53.2			*	57845			
	83.2			*	57906			
NOAC monthly drug cost	148.6			*	57084			
	399.5			*	58527			
minor ischemic stroke upfront cost	10712			*	57985			
	15000			*	57672			
moderate ischemic stroke upfront cost	15009			*	58112			
	19120			*	57811			
severe ischemic stroke upfront cost	19442			*	58123			
	32360			*	57221			
minor ischemic stroke monthly maintenance cost	570			*	58100			
	729			*	57695			
moderate ischemic stroke monthly maintenance cost	1247			*	61260			
	3463			*	54535			
severe ischemic stroke monthly maintenance cost	2355		*	13192	60079			
	7292			*	55716			
nondisabling ICH upfront cost	16646			*	57773			
	37163			*	58069			

© 2022 Chen W et al. *JAMA Health Forum.*

disabling ICH upfront cost	53526			*	56931			
	116485			*	59317			
minor bleed upfront cost	113.7			*	57883			
	162.1			*	57896			
nondisabling ICH monthly maintenance cost	1397			*	57684			
	2095			*	58103			
disabling ICH monthly maintenance cost	2502			*	57569			
	3752			*	58216			
pulse palpation cost	0.31			*	57942			
	0.85			*	57884			
12L ECG cost	32.0			*	57892			
RN visit cost	9.4			*	57838			
	25.6			*	57900			
major bleed upfront cost	6703			*	57594			
	45612			*	59865			
patch monitor cost	199			*	58024			
OAC uptake rate	60			*	66393			
	100			*	61450			

* denotes baseline condition
ICER = incremental cost-effectiveness ratio; Freq = frequency; PP = pulse palpation; 12L = 12-lead electrocardiogram; PPG = wearable photoplethysmography; 1L = wearable single-lead electrocardiogram; PM = patch monitor; AF = atrial fibrillation; RR = relative risk; OAC = oral anticoagulant; NOAC = novel oral anticoagulant

© 2022 Chen W et al. *JAMA Health Forum.*

eFigure. Cost-effectiveness Acceptability Curves

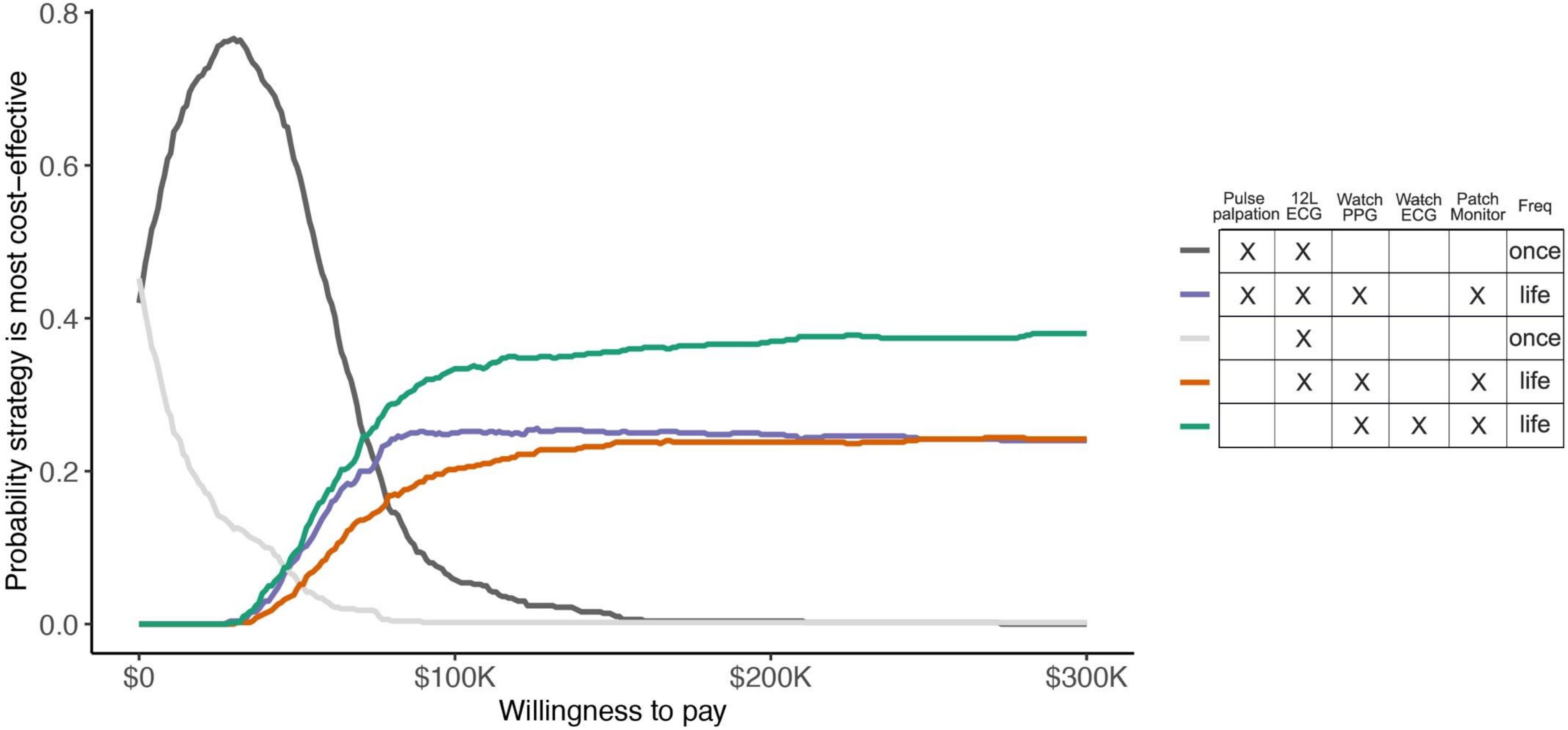

Depicted is the overall probability of greatest cost-effectiveness for specific strategies (y-axis) as a function of increasing willingness-to-pay (x-axis). Probabilities account for parameter uncertainty in probabilistic sensitivity analyses (see main text). Each colored line represents a specific screening strategy (see legend), and the highest line at a given point on the x-axis represents the strategy most likely to be cost-effective at that willingness-to-pay threshold. Strategies with probability of greatest cost-effectiveness <1% are not depicted.

© 2022 Chen W et al. *JAMA Health Forum.*

eReferences.

1. Steinhubl SR, Waalen J, Edwards AM, et al. Effect of a Home-Based Wearable Continuous ECG Monitoring Patch on Detection of Undiagnosed Atrial Fibrillation: The mSToPS Randomized Clinical Trial. *JAMA*. 2018;320(2):146-155. doi:10.1001/jama.2018.8102

2. Halcox JPJ, Wareham K, Cardew A, et al. Assessment of Remote Heart Rhythm Sampling Using the AliveCor Heart Monitor to Screen for Atrial Fibrillation: The REHEARSE-AF Study. *Circulation*. 2017;136(19):1784-1794. doi:10.1161/CIRCULATIONAHA.117.030583

3. Fitzmaurice DA, Hobbs FDR, Jowett S, et al. Screening versus routine practice in detection of atrial fibrillation in patients aged 65 or over: cluster randomised controlled trial. *BMJ*. 2007;335(7616):383. doi:10.1136/bmj.39280.660567.55

4. Rooney MR, Soliman EZ, Lutsey PL, et al. Prevalence and Characteristics of Subclinical Atrial Fibrillation in a Community-Dwelling Elderly Population: The ARIC Study. *Circ Arrhythm Electrophysiol*. 2019;12(10):e007390. doi:10.1161/CIRCEP.119.007390

5. Turakhia MP, Ziegler PD, Schmitt SK, et al. Atrial Fibrillation Burden and Short-Term Risk of Stroke: Case-Crossover Analysis of Continuously Recorded Heart Rhythm From Cardiac Electronic Implanted Devices. *Circ Arrhythm Electrophysiol*. 2015;8(5):1040-1047. doi:10.1161/CIRCEP.114.003057

6. Go AS, Reynolds K, Yang J, et al. Association of Burden of Atrial Fibrillation With Risk of Ischemic Stroke in Adults With Paroxysmal Atrial Fibrillation: The KP-RHYTHM Study. *JAMA Cardiol*. 2018;3(7):601-608. doi:10.1001/jamacardio.2018.1176

7. Diederichsen SZ, Haugan KJ, Kronborg C, et al. A Comprehensive Evaluation of Rhythm Monitoring Strategies in Screening for Atrial Fibrillation: Insights from Patients at Risk Long-Term Monitored with Implantable Loop Recorder. *Circulation*. Published online March 2, 2020:CIRCULATIONAHA.119.044407. doi:10.1161/CIRCULATIONAHA.119.044407

8. Caro JJ, Briggs AH, Siebert U, Kuntz KM, ISPOR-SMDM Modeling Good Research Practices Task Force. Modeling good research practices--overview: a report of the ISPOR-SMDM Modeling Good Research Practices Task Force-1. *Med Decis Mak Int J Soc Med Decis Mak*. 2012;32(5):667-677. doi:10.1177/0272989X12454577

9. Heeringa J, van der Kuip DAM, Hofman A, et al. Prevalence, incidence and lifetime risk of atrial fibrillation: the Rotterdam study. *Eur Heart J*. 2006;27(8):949-953. doi:10.1093/eurheartj/ehi825

10. Rothwell PM, Coull AJ, Giles MF, et al. Change in stroke incidence, mortality, case-fatality, severity, and risk factors in Oxfordshire, UK from 1981 to 2004 (Oxford

© 2022 Chen W et al. *JAMA Health Forum.*

Vascular Study). *Lancet Lond Engl*. 2004;363(9425):1925-1933. doi:10.1016/S0140-6736(04)16405-2

11. Friberg L, Rosenqvist M, Lip GYH. Evaluation of risk stratification schemes for ischaemic stroke and bleeding in 182 678 patients with atrial fibrillation: the Swedish Atrial Fibrillation cohort study. *Eur Heart J*. 2012;33(12):1500-1510. doi:10.1093/eurheartj/ehr488

12. ASCEND Study Collaborative Group, Bowman L, Mafham M, et al. Effects of Aspirin for Primary Prevention in Persons with Diabetes Mellitus. *N Engl J Med*. 2018;379(16):1529-1539. doi:10.1056/NEJMoa1804988

13. Connolly SJ, Eikelboom J, Joyner C, et al. Apixaban in patients with atrial fibrillation. *N Engl J Med*. 2011;364(9):806-817. doi:10.1056/NEJMoa1007432

14. Granger CB, Alexander JH, McMurray JJV, et al. Apixaban versus warfarin in patients with atrial fibrillation. *N Engl J Med*. 2011;365(11):981-992. doi:10.1056/NEJMoa1107039

15. Giugliano RP, Ruff CT, Braunwald E, et al. Edoxaban versus warfarin in patients with atrial fibrillation. *N Engl J Med*. 2013;369(22):2093-2104. doi:10.1056/NEJMoa1310907

16. Connolly SJ, Ezekowitz MD, Yusuf S, et al. Dabigatran versus warfarin in patients with atrial fibrillation. *N Engl J Med*. 2009;361(12):1139-1151. doi:10.1056/NEJMoa0905561

17. Patel MR, Mahaffey KW, Garg J, et al. Rivaroxaban versus warfarin in nonvalvular atrial fibrillation. *N Engl J Med*. 2011;365(10):883-891. doi:10.1056/NEJMoa1009638

18. Matsumura-Nakano Y, Shizuta S, Komasa A, et al. Open-Label Randomized Trial Comparing Oral Anticoagulation With and Without Single Antiplatelet Therapy in Patients With Atrial Fibrillation and Stable Coronary Artery Disease Beyond 1 Year After Coronary Stent Implantation. *Circulation*. 2019;139(5):604-616. doi:10.1161/CIRCULATIONAHA.118.036768

19. Zheng SL, Roddick AJ. Association of Aspirin Use for Primary Prevention With Cardiovascular Events and Bleeding Events: A Systematic Review and Meta-analysis. *JAMA*. 2019;321(3):277-287. doi:10.1001/jama.2018.20578

20. Antithrombotic Trialists' (ATT) Collaboration, Baigent C, Blackwell L, et al. Aspirin in the primary and secondary prevention of vascular disease: collaborative meta-analysis of individual participant data from randomised trials. *Lancet Lond Engl*. 2009;373(9678):1849-1860. doi:10.1016/S0140-6736(09)60503-1

21. Stroke Prevention in Atrial Fibrillation Study. Final results. *Circulation*. 1991;84(2):527-539.

© 2022 Chen W et al. *JAMA Health Forum.*

22. Sanmuganathan PS, Ghahramani P, Jackson PR, Wallis EJ, Ramsay LE. Aspirin for primary prevention of coronary heart disease: safety and absolute benefit related to coronary risk derived from meta-analysis of randomised trials. *Heart Br Card Soc*. 2001;85(3):265-271. doi:10.1136/heart.85.3.265

23. Ridker PM, Cook NR, Lee IM, et al. A randomized trial of low-dose aspirin in the primary prevention of cardiovascular disease in women. *N Engl J Med*. 2005;352(13):1293-1304. doi:10.1056/NEJMoa050613

24. Mauri L, Kereiakes DJ, Yeh RW, et al. Twelve or 30 months of dual antiplatelet therapy after drug-eluting stents. *N Engl J Med*. 2014;371(23):2155-2166. doi:10.1056/NEJMoa1409312

25. Benjamin EJ, Muntner P, Alonso A, et al. Heart Disease and Stroke Statistics-2019 Update: A Report From the American Heart Association. *Circulation*. 2019;139(10):e56-e528. doi:10.1161/CIR.0000000000000659

26. Barker WH, Mullooly JP, Getchell W. Changing incidence and survival for heart failure in a well-defined older population, 1970-1974 and 1990-1994. *Circulation*. 2006;113(6):799-805. doi:10.1161/CIRCULATIONAHA.104.492033

27. McKee PA, Castelli WP, McNamara PM, Kannel WB. The natural history of congestive heart failure: the Framingham study. *N Engl J Med*. 1971;285(26):1441-1446. doi:10.1056/NEJM197112232852601

28. Dannenberg AL, Garrison RJ, Kannel WB. Incidence of hypertension in the Framingham Study. *Am J Public Health*. 1988;78(6):676-679. doi:10.2105/ajph.78.6.676

29. Bancks MP, Kershaw K, Carson AP, Gordon-Larsen P, Schreiner PJ, Carnethon MR. Association of Modifiable Risk Factors in Young Adulthood With Racial Disparity in Incident Type 2 Diabetes During Middle Adulthood. *JAMA*. 2017;318(24):2457-2465. doi:10.1001/jama.2017.19546

30. Atherosclerosis Risk in Communities Study. https://www2.cscc.unc.edu/aric/sites/default/files/public/manuals/Updates%20Manual3_20151112.pdf. Accessed July 27, 2020.

31. Cea-Soriano L, Fowkes FGR, Johansson S, Allum AM, García Rodriguez LA. Time trends in peripheral artery disease incidence, prevalence and secondary preventive therapy: a cohort study in The Health Improvement Network in the UK. *BMJ Open*. 2018;8(1):e018184. doi:10.1136/bmjopen-2017-018184

32. Briffa T, Nedkoff L, Peeters A, et al. Discordant age and sex-specific trends in the incidence of a first coronary heart disease event in Western Australia from 1996 to 2007. *Heart Br Card Soc*. 2011;97(5):400-404. doi:10.1136/hrt.2010.210138

© 2022 Chen W et al. *JAMA Health Forum.*

33. Eagle KA, Rihal CS, Foster ED, Mickel MC, Gersh BJ. Long-term survival in patients with coronary artery disease: importance of peripheral vascular disease. The Coronary Artery Surgery Study (CASS) Investigators. *J Am Coll Cardiol*. 1994;23(5):1091-1095. doi:10.1016/0735-1097(94)90596-7

34. Inglis SC, Bebchuk J, Al-Suhaim SA, et al. Peripheral artery disease and outcomes after myocardial infarction: an individual-patient meta-analysis of 28,771 patients in CAPRICORN, EPEHESUS, OPTIMAAL and VALIANT. *Int J Cardiol*. 2013;168(2):1094-1101. doi:10.1016/j.ijcard.2012.11.033

35. Bonaca MP, Bhatt DL, Storey RF, et al. Ticagrelor for Prevention of Ischemic Events After Myocardial Infarction in Patients With Peripheral Artery Disease. *J Am Coll Cardiol*. 2016;67(23):2719-2728. doi:10.1016/j.jacc.2016.03.524

36. Attar R, Wester A, Koul S, Eggert S, Andell P. Peripheral artery disease and outcomes in patients with acute myocardial infarction. *Open Heart*. 2019;6(1):e001004. doi:10.1136/openhrt-2018-001004

37. Tunstall-Pedoe H, Peters SAE, Woodward M, Struthers AD, Belch JJF. Twenty-Year Predictors of Peripheral Arterial Disease Compared With Coronary Heart Disease in the Scottish Heart Health Extended Cohort (SHHEC). *J Am Heart Assoc*. 2017;6(9). doi:10.1161/JAHA.117.005967

38. Hiatt WR, Fowkes FGR, Heizer G, et al. Ticagrelor versus Clopidogrel in Symptomatic Peripheral Artery Disease. *N Engl J Med*. 2017;376(1):32-40. doi:10.1056/NEJMoa1611688

39. Burn J, Dennis M, Bamford J, Sandercock P, Wade D, Warlow C. Long-term risk of recurrent stroke after a first-ever stroke. The Oxfordshire Community Stroke Project. *Stroke*. 1994;25(2):333-337.

40. Yung D, Kapral MK, Asllani E, Fang J, Lee DS, Investigators of the Registry of the Canadian Stroke Network. Reinitiation of anticoagulation after warfarin-associated intracranial hemorrhage and mortality risk: the Best Practice for Reinitiating Anticoagulation Therapy After Intracranial Bleeding (BRAIN) study. *Can J Cardiol*. 2012;28(1):33-39. doi:10.1016/j.cjca.2011.10.002

41. Hylek EM, Go AS, Chang Y, et al. Effect of intensity of oral anticoagulation on stroke severity and mortality in atrial fibrillation. *N Engl J Med*. 2003;349(11):1019-1026. doi:10.1056/NEJMoa022913

42. Marini C, De Santis F, Sacco S, et al. Contribution of atrial fibrillation to incidence and outcome of ischemic stroke: results from a population-based study. *Stroke*. 2005;36(6):1115-1119. doi:10.1161/01.STR.0000166053.83476.4a

43. Anderson CS, Jamrozik KD, Broadhurst RJ, Stewart-Wynne EG. Predicting survival for 1 year among different subtypes of stroke. Results from the Perth Community Stroke Study. *Stroke*. 1994;25(10):1935-1944.

© 2022 Chen W et al. *JAMA Health Forum.*

44. van Asch CJ, Luitse MJ, Rinkel GJ, van der Tweel I, Algra A, Klijn CJ. Incidence, case fatality, and functional outcome of intracerebral haemorrhage over time, according to age, sex, and ethnic origin: a systematic review and meta-analysis. *Lancet Neurol*. 2010;9(2):167-176. doi:10.1016/S1474-4422(09)70340-0

45. Rosand J, Eckman MH, Knudsen KA, Singer DE, Greenberg SM. The effect of warfarin and intensity of anticoagulation on outcome of intracerebral hemorrhage. *Arch Intern Med*. 2004;164(8):880-884. doi:10.1001/archinte.164.8.880

46. Hardie K, Hankey GJ, Jamrozik K, Broadhurst RJ, Anderson C. Ten-year survival after first-ever stroke in the perth community stroke study. *Stroke*. 2003;34(8):1842-1846. doi:10.1161/01.STR.0000082382.42061.EE

47. McNeil JJ, Wolfe R, Woods RL, et al. Effect of Aspirin on Cardiovascular Events and Bleeding in the Healthy Elderly. *N Engl J Med*. 2018;379(16):1509-1518. doi:10.1056/NEJMoa1805819

48. Landefeld CS, Beyth RJ. Anticoagulant-related bleeding: clinical epidemiology, prediction, and prevention. *Am J Med*. 1993;95(3):315-328.

49. Yong JHE, Thavorn K, Hoch JS, et al. Potential Cost-Effectiveness of Ambulatory Cardiac Rhythm Monitoring After Cryptogenic Stroke. *Stroke*. 2016;47(9):2380-2385. doi:10.1161/STROKEAHA.115.011979

50. Neyt M, Van Brabandt H, Devos C. The cost-utility of catheter ablation of atrial fibrillation: a systematic review and critical appraisal of economic evaluations. *BMC Cardiovasc Disord*. 2013;13:78. doi:10.1186/1471-2261-13-78

51. Sullivan PW, Arant TW, Ellis SL, Ulrich H. The cost effectiveness of anticoagulation management services for patients with atrial fibrillation and at high risk of stroke in the US. *PharmacoEconomics*. 2006;24(10):1021-1033. doi:10.2165/00019053-200624100-00009

52. Gage BF, Cardinalli AB, Owens DK. The effect of stroke and stroke prophylaxis with aspirin or warfarin on quality of life. *Arch Intern Med*. 1996;156(16):1829-1836.

53. O'Brien CL, Gage BF. Costs and effectiveness of ximelagatran for stroke prophylaxis in chronic atrial fibrillation. *JAMA*. 2005;293(6):699-706. doi:10.1001/jama.293.6.699

54. Flaker GC, Belew K, Beckman K, et al. Asymptomatic atrial fibrillation: demographic features and prognostic information from the Atrial Fibrillation Follow-up Investigation of Rhythm Management (AFFIRM) study. *Am Heart J*. 2005;149(4):657-663. doi:10.1016/j.ahj.2004.06.032

55. Harrington Amanda R., Armstrong Edward P., Nolan Paul E., Malone Daniel C. Cost-effectiveness of Apixaban, Dabigatran, Rivaroxaban, and Warfarin for Stroke

© 2022 Chen W et al. *JAMA Health Forum.*

Prevention in Atrial Fibrillation. *Stroke*. 2013;44(6):1676-1681. doi:10.1161/STROKEAHA.111.000402

56. Centers for Medicare & Medicaid Services. https://www.cms.gov

57. Agency for Healthcare Research and Quality. https://hcupnet.ahrq.gov/#setup

58. Yong Jean Hai Ein, Thavorn Kednapa, Hoch Jeffrey S., et al. Potential Cost-effectiveness of Ambulatory Cardiac Rhythm Monitoring After Cryptogenic Stroke. *Stroke*. 2016;47(9):2380-2385. doi:10.1161/STROKEAHA.115.011979

59. Lowres N, Neubeck L, Salkeld G, et al. Feasibility and cost-effectiveness of stroke prevention through community screening for atrial fibrillation using iPhone ECG in pharmacies. *Thromb Haemost*. 2014;111(6):1167-1176. doi:10.1160/TH14-03-0231

60. Oguz M, Lanitis T, Li X, et al. Cost-effectiveness of Extended and One-Time Screening Versus No Screening for Non-Valvular Atrial Fibrillation in the USA. *Appl Health Econ Health Policy*. 2020;18(4):533-545. doi:10.1007/s40258-019-00542-y

61. Medical Expenditure Panel Survey (MEPS), Agency for Healthcare Research and Quality. https://meps.ahrq.gov/about_meps/Price_Index.shtml#t2a2

62. U.S. Bureau of Labor Statistics. https://data.bls.gov/cgi-bin/surveymost?bls

© 2022 Chen W et al. *JAMA Health Forum.*
